# Nitrogen Functionalities of Amino-Functionalized Nitrogen-Doped Graphene Quantum Dots for Highly Efficient Enhancement of Antimicrobial Therapy to Eliminate Methicillin-Resistant *Staphylococcus aureus* and Utilization as a Contrast Agent

**DOI:** 10.3390/ijms22189695

**Published:** 2021-09-07

**Authors:** Wen-Shuo Kuo, Ping-Ching Wu, Chi-Yao Hung, Chia-Yuan Chang, Jiu-Yao Wang, Pei-Chi Chen, Miao-Hsi Hsieh, Sheng-Han Lin, Chan-Chi Chang, Yen-Sung Lin

**Affiliations:** 1 School of Chemistry and Materials Science, Nanjing University of Information Science and Technology, Nanjing 210044, China; wskuo88@gmail.com; 2 State Key Laboratory for Chemistry and Molecular Engineering of Medicinal Resources, Guangxi Normal University, Guilin 541004, China; 3 Allergy & Clinical Immunology Research Center, National Cheng Kung University Hospital, College of Medicine, National Cheng Kung University, Tainan 701, Taiwan; a122@mail.ncku.edu.tw (J.-Y.W.); simple48686@gmail.com (P.-C.C.); karinadrift@gmail.com (M.-H.H.); 4 Allergy Immunology and Microbiome Center, China Medical University Children’s Hospital, China Medical University, Taichung 404, Taiwan; 5 Department of Biomedical Engineering, National Cheng Kung University, Tainan 701, Taiwan; wbcxyz@bme.ncku.edu.tw; 6 Institute of Oral Medicine and Department of Stomatology, National Cheng Kung University Hospital, College of Medicine, National Cheng Kung University, Tainan 701, Taiwan; 7 Center of Applied Nanomedicine, National Cheng Kung University, Tainan 701, Taiwan; 8 Medical Device Innovation Center, Taiwan Innovation Center of Medical Devices and Technology, National Cheng Kung University Hospital, College of Medicine, National Cheng Kung University, Tainan 701, Taiwan; 9 Department of Physical Medicine and Rehabilitation, An Nan Hospital, China Medical University, Tainan 709, Taiwan; chiyaohung05@gmail.com; 10 Department of Mechanical Engineering, National Cheng Kung University, Tainan 701, Taiwan; cychang0829@gs.ncku.edu.tw; 11 Department of Anesthesiology, E-Da Hospital, Kaohsiung 824, Taiwan; 12 Department of Otolaryngology, National Cheng Kung University Hospital, College of Medicine, National Cheng Kung University, Tainan 701, Taiwan; 13 Division of Pulmonary and Critical Care Medicine, An Nan Hospital, China Medical University, Tainan 709, Taiwan; 14 Department of Nursing, Chung Hwa University of Medical Technology, Tainan 717, Taiwan

**Keywords:** graphene quantum dot with nitrogen doping and amino group functionalization, photodynamic therapy, multidrug-resistant methicillin-resistant *Staphylococcus aureus*, reactive oxygen species, contrast probe

## Abstract

There is an urgent need for materials that can efficiently generate reactive oxygen species (ROS) and be used in photodynamic therapy (PDT) as two-photon imaging contrast probes. In this study, graphene quantum dots (GQDs) were subjected to amino group functionalization and nitrogen doping (amino-N-GQDs) via annealing and hydrothermal ammonia autoclave treatments. The synthesized dots could serve as a photosensitizer in PDT and generate more ROS than conventional GQDs under 60-s low-energy (fixed output power: 0.07 W·cm^−2^) excitation exerted by a 670-nm continuous-wave laser. The generated ROS were used to completely eliminate a multidrug-resistant strain of methicillin-resistant *Staphylococcus aureus* (MRSA), a Gram-positive bacterium. Compared with conventional GQDs, the amino-N-GQDs had superior optical properties, including stronger absorption, higher quantum yield (0.34), stronger luminescence, and high stability under exposure. The high photostability and intrinsic luminescence of amino-N-GQDs contribute to their suitability as contrast probes for use in biomedical imaging, in addition to their bacteria tracking and localization abilities. Herein, the dual-modality amino-N-GQDs in PDT easily eliminated multidrug-resistant bacteria, ultimately revealing their potential for use in future clinical applications.

## 1. Introduction

Materials that are primarily based on graphene have exceptional chemical stability and mechanical, thermal, and electronic properties. Thus, these materials have great potential for use in nanodevices. However, currently available graphene-based materials produced by typical physical and chemical routes, including micromechanical cleavage, reduction in exfoliated graphene oxide, and solvothermal synthesis, are generally micrometer-sized graphene sheets, limiting their direct applications in nanodevices [1]. As a result, there is an urgent need to develop effective routes for cutting large graphene sheets into nanometer-sized pieces with a well-confined shape, such as graphene nanoribbons and graphene quantum dots (GQDs) [2]. GQDs are a novel class of carbon materials that are smaller than 10 nm. They were first reported in a 2004 study where single-walled carbon nanotubes were purified through preparative electrophoresis [3]. GQDs have become increasingly crucial members of the nanocarbon family owing to their benignity, abundance, and low cost. Carbon is usually black and has been recently thought to have weak fluorescence and low solubility in water [4]. As GQDs exhibit strong fluorescence, they have attracted considerable attention, attaining the moniker “fluorescent carbons” [5]. Compared with conventional organic dyes, photoluminescent GQDs possess superior properties, such as aqueous solubility, easy functionalization, high resistance to photobleaching, excellent chemical inertness, high biocompatibility, and low toxicity [6]. Consequently, their potential for use in biological labeling, bioimaging, and drug delivery has been widely explored. GQDs have been found to emit visible to near-infrared (NIR) photoluminescence (PL) under light excitation, which is of particular interest. NIR PL emissions from GQDs excited using NIR light are particularly critical for bionanotechnology applications due to body tissue transparency in the NIR window for water [7]. PL emissions from GQDs can be efficiently quenched using electron acceptors or donor molecules in solution, which reveal the excellent electron acceptance and donation abilities of photoexcited GQDs. Owing to their notable photoinduced electron transfer properties, GQDs may be suitable for use in photovoltaic devices and light energy conversion, among other applications [8]. GQDs are also promising nanoprobes for sensitive detection [9].

Doping is crucial for semiconductors as it can considerably change the carrier density of a material and, thus, completely alter its intrinsic electrical and optical properties [10,11]. Nitrogen (N) doping or functionalization of GQDs is an extremely helpful method for altering the intrinsic properties of GQDs. N atoms, with atomic sizes comparable to those of carbon atoms, have five valence electrons and are highly accepting of electrons; thus, they provide the adjacent carbon atoms in graphene with a comparatively high level of positive charge density. In molecular systems with an sp^2^ bond, carbon can be replaced with nitrogen, which can enable the formation of heterocyclic aromatic compounds. The inherent features of materials that are based on carbon, including their electronic characteristics and local and surface chemical features, can be effectively modified by atom doping. N-atom doping of GQDs (size < 10 nm), which exhibit notable quantum confinement and edge effects, results in the modulation of the dots’ chemical composition and bandgap. Because the properties of N-doped GQDs (N-GQDs) can be modulated, these dots should exhibit improved electrochemical, electrocatalytic, and photochemical activity and possess tunable luminescence, which is critical for optoelectronic and bioimaging applications [12]. Accordingly, chemical doping can be considered an effective approach for tailoring the optical, chemical, and electronic properties of graphene. GQDs subjected to amino group functionalization and nitrogen doping (amino-N-GQDs) have electronic properties strongly influenced by chemical modifications that enable strong electron donation in primary amine molecules (a phenomenon referred to as amino group functionalization) [13]. When amino-N-GQDs undergo singlet–triplet splitting, the corresponding intersystem crossing is sufficiently efficient to compete with internal conversion between multiplicity-identical states, which results in simultaneous PL and the creation of reactive oxygen species (ROS) involved in photodynamic therapy (PDT) [14].

In PDT, ROS are formed when molecular oxygen reacts with a photoexcited photosensitizer (PS) exposed to a suitable wavelength of light source and energy [15]. Photosensitized reactions involving oxygen are categorized as type I or II. A light-sensitized (excited) PS can directly react with a suitable substrate (unsaturated lipids, proteins, or nucleic acids) to produce unstable radicals through proton or electron transfer (type I reaction), leading to oxygenated products in the presence of oxygen, such as a superoxide anion radical (O_2_^−^), hydroxyl radicals (OH.), or hydrogen peroxide (H_2_O_2_). Then, they react with molecular oxygen to form singlet oxygen (^1^O_2_) through energy transfer (type II reaction) [16]. Cells or bacteria can be irreversibly damaged when ROS undergo oxidative reactions with adjacent biological substrates. In addition to the application of conventional and newly synthesized PSs, the use of materials for PDT is a potentially favorable approach for improving the efficacy of therapy. However, because materials are used in combination with PSs, some studies have reported the induction of PDT mechanisms [11,14]. Few studies have directly employed amino-N-GQDs as PSs for ROS generation in PDT [11,14]. Accordingly, in the present study, amino-N-GQDs were employed in PDT as PSs to eliminate a multidrug-resistant (MDR) strain of methicillin-resistant *Staphylococcus aureus* (MRSA), a Gram-positive bacterium, using a 670-nm continuous-wave laser. A low concentration (0.5 μg·mL^−1^) of amino-N-GQDs and a photoexcitation time of 60 s with an energy irradiation dose of 0.07 W·cm^−2^ were found to result in the elimination of all bacteria [17,18]. However, using GQDs instead of amino-N-GQDs in the same treatment resulted in 37% bacterial elimination. The properties of the PL (Em: 696 nm)—optimal irradiation penetration, low energy absorption, and slight scattering—emitted in the NIR region revealed that the derived amino-N-GQDs are promising contrast probes for tracking and localizing amino-N-GQD-treated bacteria and could provide further information regarding the status of the irradiated bacteria. The combination of dual-modality PDT, a contrast agent, and amino-N-GQDs was determined to be promising for eliminating and tracking MDR bacteria.

## 2. Results and Discussion

**Figure 1:**
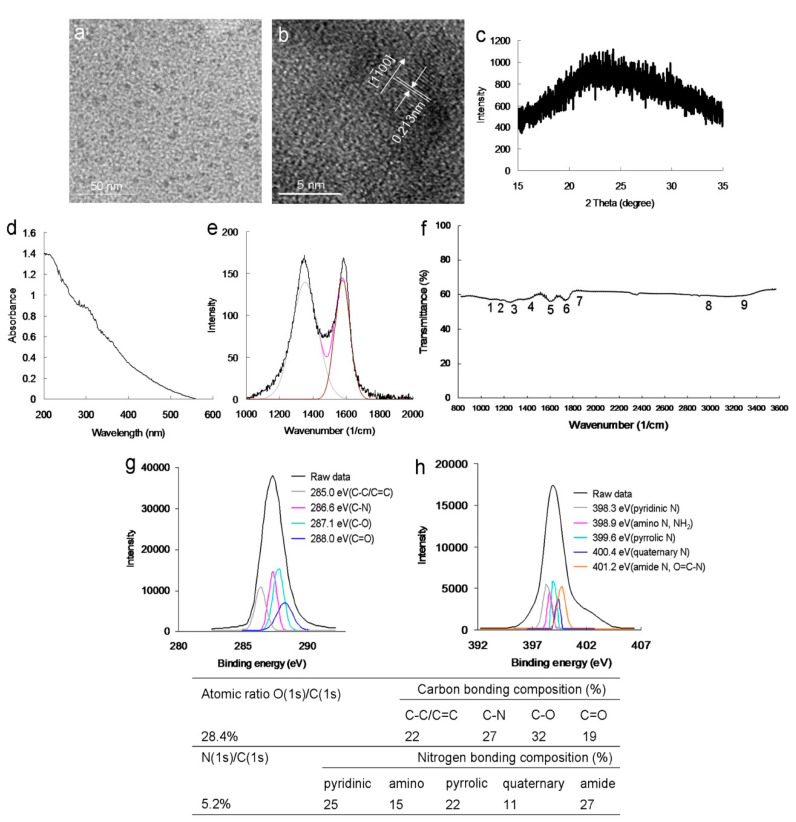
(**a**) Low-magnified transmission electron microscopy (TEM) image; (**b**) High-resolution (HR) TEM image captured for a single amino-N-graphene quantum dot (GQD) (mean lateral size = ~8.3 nm; interlayer spacing = 0.213 nm); the size distribution of its histogram was determined using dynamic light scattering (DLS) (Figure S1a). (**c**) X-ray diffraction (XRD) pattern of amino-N-GQDs. (**d**) Raman spectrum of amino-N-GQDs, with the gray and brown lines representing the spectrum decomposed and fitted to the D- and G-band peaks (at ~1384 and 1606 cm^−1^, respectively; black line: raw data; pink line: decomposed spectrum). (**e**) Ultraviolet-visible (UV-vis), and (**f**) Fourier-transform infrared (FTIR) spectra of amino-N-GQDs. (**g**,**h**) X-ray photoelectron spectroscopy (XPS) spectrum of amino-N-GQDs obtained to determine the changes in the chemical state. In the deconvoluted C(1s) spectrum, the peaks were fitted using the Gaussian function corresponding to C–C/C=C, C–N, C–O, and C=O. In the deconvoluted N(1s) spectrum, the peaks were fitted using the Gaussian function corresponding to pyridinic N, amino N, pyrrolic N, quaternary N, and amide N. The table summarizes the bonding composition and atomic ratio of the amino-N-GQDs. The O(1s)/C(1s) and N(1s)/C(1s) atomic ratios were 28.4% and 5.2%, respectively. Delivered dose: 0.5 μg·mL^−1^ to 5 mg·mL^−1^ material.

The modified Hummers method [19] was employed to prepare a graphene oxide sheet, which was subjected to an ultrasonic shearing reaction to synthesize amino-N-GQDs [20]. The mean lateral size of the amino-N-GQDs was approximately 8.3 nm (Figure S1a), as determined using low-magnification (Figure 1a) and high-resolution transmission electron microscopy (HR-TEM) images (Figure 1b). The interlayer spacing of the as-prepared amino-N-GQDs was 0.213 nm, which corresponded to the *d*-spacing of the {$1\overset{-}{1}$00} lattice planes of graphene (Figure 1b) [6]. Figure 1c displays the crystallinity analysis-derived X-ray diffraction (XRD) spectrum. The diffraction angle of the amino-N-GQD material was nearly 2*θ* = 24.3°, indicating appropriate layer regularity as well as a well-ordered lamellar structure exhibiting a 0.360-nm interlayer distance. The interfringe distances were 0.213 and 0.360 nm, which correspond to the in-plane lattice spacing and basal plane distance of graphite, respectively. The values for amino-N-GQDs are consistent with those reported previously (0.340–0.403 nm) [5]. However, based on the XRD findings, the basal plane was not considerably functionalized. This finding aligns with the theory that, compared with in-plane carbons, graphene has considerably more active edges, and functional groups are present at the amino-N-GQD edges [15]. The large basal spacing of the amino-N-GQDs is due to the accommodation of several oxygen species, such as epoxy, hydroxyl, and amino groups, and alteration of the carbon hexahedron grid plane, resulting in greater graphene layer spacing. Various peaks were present in the UV-vis absorption spectrum of the amino-N-GQDs, specifically at 315 nm (*n* → π* transition of the C=O shoulder and C–N) and 212 nm (*π* → π* transition of the aromatic C=C bonds; Figure 1d). The absorption peaks in the spectrum of amino-N-GQDs containing oxygen corresponded to the *π* electron transition and revealed that the GQDs were successfully doped with N. The crystallinity of the amino-N-GQDs was investigated using Raman spectroscopy. The peak at 1384 cm^−1^ (D band) was identified to be associated with the vibration of the sp3-carbon atoms in disordered graphite, while the peak at 1606 cm^−1^ (G band) corresponded with the vibration of the sp2 hybrid carbon atoms. Further, the integrated D to G band intensity ratio (*I*_D_/*I*_G_), which indicates the degree of graphitization, was approximately 0.90, indicating that the amino-N-GQDs were highly distorted (Figure 1e) [21]. The *I*_D_/*I*_G_ ratios were used as inputs in the Raman calculations to determine the mean sp^2^ domain size of the GQD-based specimens [22]. The estimated size almost matched the one obtained from HR-TEM, although the value obtained from the Raman calculations (~8.0 nm) was slightly lower due to the Raman estimation (Equations (S2) and (S3)) [22]. The exposed functional groups of the amino-N-GQDs were directly analyzed using Fourier-transform infrared (FTIR) spectroscopy, revealing characteristic bands corresponding to C–O stretching at approximately 1023 cm^−1^ (band 1), C–N stretching at approximately 1150 cm^−1^ (band 2), N–C=O stretching at approximately 1212 cm^−1^ (band 3), tertiary alcohol C–OH bonding at approximately 1448 cm^−1^ (band 4), a C=C ring at approximately 1611 cm^−1^ (band 5), N–H bonding and amide at approximately 1786 cm^−1^ (band 6), C=O stretching at approximately 1833 cm^−1^ (band 7), N–H stretching at approximately 2971 cm^−1^ (band 8), and N–H vibration at approximately 3455 cm^−1^ (band 9), ultimately indicating the carbonyl, hydroxyl, and amino groups exposed on the material (Figure 1f). Because of these exposed functional groups, the amino-N-GQDs had a surface charge of 16.4 mV (in ddH_2_O) based on the zeta potential findings. X-ray photoelectron spectroscopy (XPS) was also performed to evaluate the surface chemistry of the amino-N-GQDs. The deconvoluted C(1s) spectrum indicated a non-oxygenated ring (C–C/C=C, 285.0 eV), C–N (286.6 eV), C–O (287.1 eV), and carbonyl (C=O, 288.0 eV) bonds (Figure 1g). Moreover, the deconvoluted N(1s) spectrum indicated pyridinic N (398.3 eV), amino N (NH_2_, 398.9 eV), pyrrolic N (399.6 eV), quaternary N (400.4 eV), and amide N (O=C–N, 401.2 eV; Figure 1h). The table in Figure 1 summarizes the bonding composition and atomic ratio of amino-N-GQDs. The characterization results indicated the successful preparation of amino-N-GQDs.

**Figure 2:**
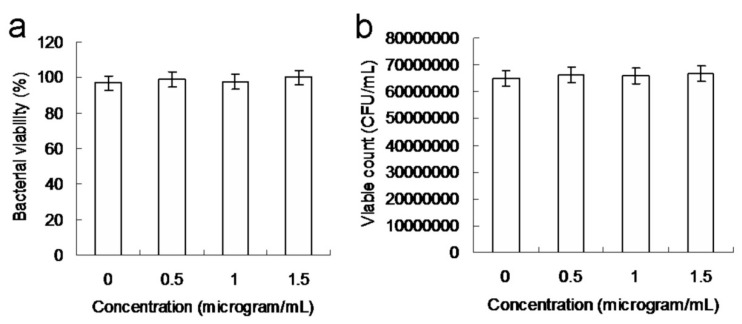
Number of surviving (**a**) amino-N-GQD–Ab_protein A_-treated methicillin-resistant *Staphylococcus aureus* (MRSA) cells (expressed as a percentage) based on the colony-forming unit (CFU) counting assay, and (**b**) MRSA cells (unit: CFU/mL). Delivered dose: OD_600_ = ~0.05 μg·mL^−1^ for bacteria and 0–1.5 μg·mL^−1^ for material–Ab_protein__A_. Data are presented as mean ± SD (*n* = 6).

The continuous increase in MDR bacterial strains is a serious medical problem due to their ability to develop resistance to antibiotics through different pathways [23]. Because of the growing resistance of the Gram-positive bacteria, MRSA, to conventional antimicrobial agents, it is desirable to develop alternative approaches to eliminate MDR bacterial strains. This study employed an experimental MRSA template. The surface protein, protein A, on the cell wall of MRSA was considered. Accordingly, the material was coated with an Ab_protein A_ antibody to form the material, Ab_protein A_ hybrid, assuming it would possess enhanced specificity, selectivity, and efficiency. A colony-forming unit (CFU) assay was performed to examine material biocompatibility. The number of bacterial cells was calculated as log_10_ CFU per milliliter (log_10_ CFU/mL) and is expressed as a percentage (Figure 2a,b). The CFU assay revealed that the amino-N-GQDs were highly biocompatible. Additionally, the toxicity of the material was found to contribute to the elimination of bacteria in PDT.

The experiment would have been compromised if the amino-N-GQD samples were exposed to white light. To prevent the possibility of confounding the PDT process by inadvertently exposing the amino-N-GQD samples to white light, experiments related to PDT were conducted in the dark. Indeed, the viabilities of amino-N-GQD (0.5–1.5 μg·mL^−1^)-treated MRSA decreased from approximately 7% to 15%, which was somewhat influenced by light illumination (not in the dark) (Figure S2a,b). A low dose (0.5 μg·mL^−1^) of amino-N-GQDs that had been incubated for 3 h with MRSA (OD_600_: ~0.05) at 37 °C in the dark was employed in all subsequent experiments.

**Figure 3:**
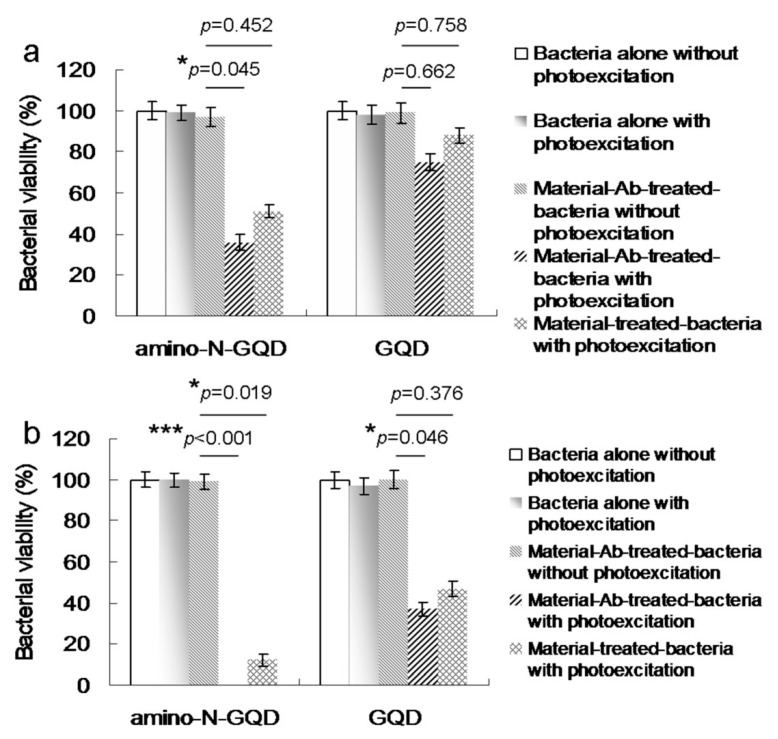
MRSA viability (%) based on the viable count of material–Ab_protein A_-treated MRSA obtained using the CFU assay under a short 670-nm laser excitation (0.07 W·cm^−2^) for (**a**) 30 and (**b**) 60 s. Delivered dose: OD600 = ~0.05 μg·mL^−1^ for bacteria and 0.5 μg·mL^−1^ for material–Ab_protein A_. Data are presented as mean ± SD (*n* = 6). For amino-N-GQD- and GQD–Ab_protein A_-treated MRSA with photoexcitation, (**a**) *p* = 0.045 and *p* = 0.662, and (**b**) *p* < 0.001 and *p* = 0.046, respectively. For amino-N-GQD- and GQD-treated MRSA with photoexcitation, (**a**) *p* = 0.425 and *p* = 0.758, and (**b**) *p* = 0.019 and *p* = 0.376, respectively. * *p* value was calculated using the Student’s *t* test (* *p* < 0.05, ** *p* < 0.01, *** *p* < 0.001).

**Figure 4:**
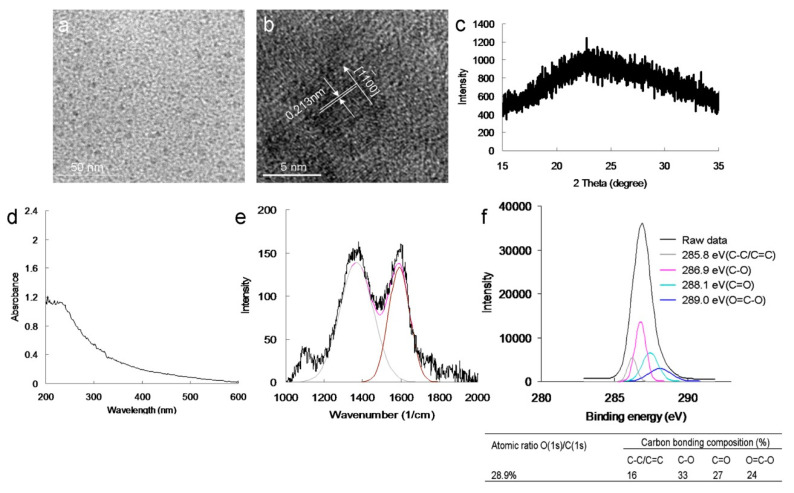
(**a**) Low-magnified TEM image; (**b**) HR-TEM image of a single GQD exhibiting the graphene {$1\overset{-}{1}$00} lattice plane (mean lateral size = 8.0 nm; *d*-spacing = 0.213 nm); the size distribution of its histogram was determined using DLS (Figure S1b). (**c**) XRD spectrum of GQDs, indicating a diffraction angle 2*θ* of nearly 24.3° and revealing a well-ordered lamellar structure with favorable layer regularity and an interlayer distance of 0.360 nm. (**d**) UV-vis spectrum of GQDs. The peak at 211 nm corresponds to the *π* → π* transition of aromatic C=C bonds, whereas that at 312 nm corresponds to the *n* → π* transitions of the C=O shoulder. (**e**) Raman spectrum of GQDs. The gray and brown lines indicate the spectrum decomposed and fitted to D- and G-band peaks (at ~1383 and 1607 cm^−1^, respectively; black line: raw data; pink line: decomposed spectrum). The *I*D/*I*G integrated intensity ratio was 0.89. The diameter obtained from the Raman calculations (Equations (S2) and (S3)) was approximately 7.8 nm. The estimated size almost matched that obtained from the HR-TEM calculations. (**f**) Deconvoluted C(1s) XPS spectra with Gaussian function-fitted peaks: nonoxygenated ring (C–C/C=C, 285.8 eV), hydroxyl (C–O, 286.9 eV), carbonyl (C=O, 288.1 eV), and carboxylate (O=C–O, 289.0 eV) groups. The table summarizes the bonding composition and atomic ratio of the GQDs. Delivered dose: from 0.5 μg·mL^−1^ to 5 mg·mL^−1^ material.

To evaluate the antimicrobial potential of the material, PDT experiments were conducted against MRSA using low-dose energy irradiation (0.07 W·cm^−2^) with an exposure time of 0–60 s. In addition, the effect of the photoexcited material, Ab_protein A_, on the viability of MRSA was determined. No bactericidal effects were observed for MRSA alone, with or without laser exposure, or on the panel of material-treated bacteria when laser treatment was not applied (Figure 3a). After photoexcitation for 30 s, the viability of amino-N-GQD–Ab_protein A_ was relatively low (approximately 36%); this value corresponded to a reduction of approximately 0.449 log_10_ (Figure S2a). In contrast, the bacterial viability was higher in the panel treated with amino-N-GQDs (51%, approximately 0.031 log_10_ reduction; Figure S3a) without antibody coating. This finding indicates that the Ab_protein A_ antibody coated the material, resulting in greater selectivity and specificity. After 60 s, the amino-N-GQD–Ab_protein A_-treated bacteria were completely eliminated (100% elimination; Figure 3b). Subsequently, we investigated whether bacterial viability was affected by N-bonding composition and amino functional groups. In identical photoexcitation experiments, the amino-N-free GQDs (characterizations, Figure 4; Figures S1b, S2a,b, S4 and S5; the characteristics of single GQDs and amino-N-GQDs were compared and are shown in Table S1) had lower bactericidal ability than the amino-N-GQDs (Figure 3). Specifically, viability levels of 75% and 37% (~0.114 and ~0.429 log_10_ reductions, respectively; Figure S3b) were observed for the GQD–Ab_protein A_-treated MRSA after different laser exposure times. Furthermore, because lipopolysaccharide (LPS) is a major component of the outer membrane of Gram-negative *Escherichia coli* (*E. coli*), GQD-Ab_LPS_ and amino-N-GQD-Ab_LPS_ also eliminated *E. coli* (Figures S2c,d, S6 and S7), exhibiting a trend similar to that shown in Figure 3.

The different incubation times had no influence on the bacterial viability; however, the viability level visibly decreased with dose (Table 1). Based on the results, the amino-N-GQDs performed PDT more efficiently than amino-N-free GQDs, amino-group-free N-GQDs, and amino-GQDs, indicating their superior efficacy. With respect to the amino-N-GQD-eliminated bacteria, which were subjected to the same treatment procedure, the experiments with the nitrogen and amino functional groups demonstrated superior bactericidal capability compared with those without these compositions.

**Figure 5:**
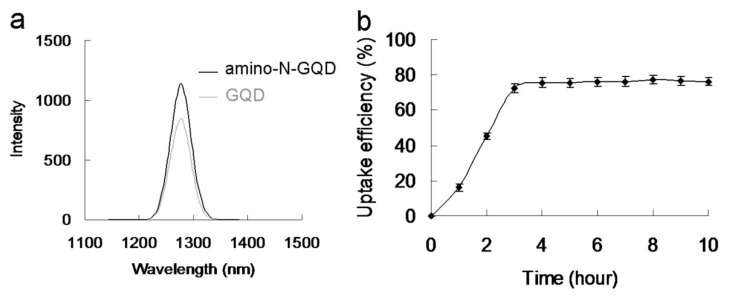
(**a**) Phosphorescence spectra measured at 1270 nm for amino-N-GQDs and GQDs. Delivered dose: 0.5 μg·mL^−1^ for material. (**b**) Uptake assay of MRSA and amino-N-GQD–Ab_protein A_, conducted at 37 °C for 10 h. Delivered dose: OD_600_ = ~0.05 μg·mL^−1^ for bacteria and 0.5 μg·mL^−1^ for amino-N-GQD–Ab_protein A_. Data are presented as mean ± SD (*n* = 6).

The aforementioned results correspond to the generated ROS, which originate from the PSs of the materials and may be influential in PDT. The oxidative stress of ROS may contribute to failure in the normal redox reaction functioning of the biological substrates surrounding bacteria treated with photoexcited material and may result in damage to DNA, ultimately causing death of the bacteria. Therefore, ^1^O_2_ and O_2_^−^ involved in PDT (photoexcited using a 670-nm laser) were detected. The intensity of ^1^O_2_ was measured by monitoring the fluorescence intensity of the singlet oxygen sensor green (SOSG) reagent and trans-1-(2′-methoxyvinyl)pyrene (*t*-MVP). Moreover, the intensity of O_2_^−^ was detected using the 470-nm absorbance of 2,3-bis (2-methoxy-4-nitro-5-sulfophenyl)-2H-tetrazolium-5-carboxanilide (XTT) and the absorbance of *γ*-l-glutamyl-l-cysteinyl-glycine (GSH) after the bacteria were incubated with the material [15,17]. The materials generated ^1^ O_2_ and O_2_^−^ in a photoexcitation time-dependent manner (photoexcitation time, 0–60 s; Table S2). However, false-positive ROS signals may have occurred due to material–ROS reagent interaction (ROS reagents included SOSG reagent, *t*-MVP, XTT, and GSH), and these false positives may have compromised the results [24]. Therefore, material–Ab_protein A_-treated MRSA was introduced and monitored (Table 2) to detect the amount of ROS formed from the laser-irradiated material. The results revealed a trend similar to that shown in Table S2, and that of material Ab_LPS_-treated *E. coli* (Table S3). However, the amino-N-GQDs were associated with greater ROS formation capability than the standard GQDs, regardless of the detection method. The amount of generated ROS was reduced after treatment with MRSA and *E. coli* without antibody coating (Tables S4 and S5). In addition, the antibody was successfully coated on the material, and its functions were performed favorably. The material demonstrated its potential application in PDT because of its notable antibacterial effect. To confirm that ROS were involved in the PDT effect of amino-N-GQDs, ROS neutralization was achieved using *α*-tocopherol [25]. The quantity of ROS decreased after the addition of *α*-tocopherol, and viability increased, as expected. After this experiment, amino-N-free GQDs were observed using XPS (Figure 4f). The amino-N-free GQDs were less capable of forming ^1^ O_2_ and O_2_^−^ than amino-N-GQDs (Table 2 and Tables S2–S5). This result is consistent with the ^1^O_2_ phosphorescence signal emitted by the material at 1270 nm (Figure 5a). The amino-N-GQDs generated greater amounts of ^1^ O_2_ and O_2_^−^ than amino-N-free GQDs. Moreover, the amino-N-GQDs had a higher Φ_Δ_ (~0.55) than the GQDs (~0.29; for reference, Φ_Δ_ = 0.64 is the quantum yield (QY) of meso-tetra [4-sulfonatophenyl] porphine dihydrochloride dissolved in D_2_O [18]).

**Figure 6:**
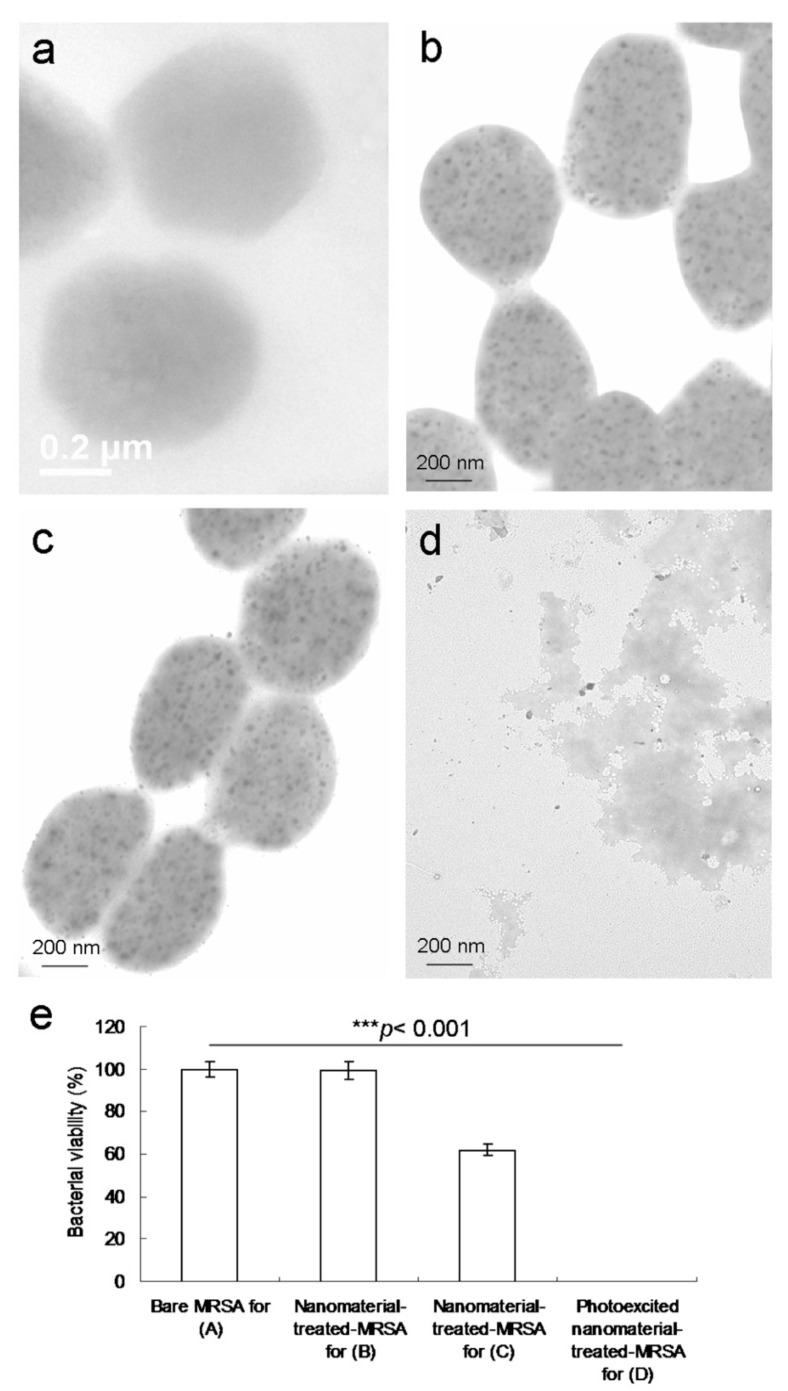
TEM images of (**a**) untreated MRSA; amino-N-GQD–Ab_protein A_-treated bacteria after (**b**) 3 h and (**c**) 4 days of incubation; (**d**) material–Ab-treated bacteria after photoexcitation (0.07 W cm^−2^; Ex: 670 nm) for 60 s followed by 3 h of additional incubation to effectively process the photodynamic therapy (PDT) action. (**e**) MRSA viability (%) based on the viable count of material–Ab_protein A_-treated MRSA obtained using the CFU assay in a short excitation period with the same treatment. * *p* value obtained using the Student’s *t* test (* *p* < 0.05, ** *p* < 0.01, *** *p* < 0.001). Delivered dose: OD_600_ = ~0.05 μg·mL^−1^ for bacteria and 0.5 μg·mL^−1^ for material–Ab_protein A_. Data are presented as mean ± SD (*n* = 6).

The aforementioned results indicate that N atoms can replace carbon atoms (through atom doping) in sp^2^-bonded molecular systems, generating heterocyclic aromatic compounds. Doping is, thus, a productive approach that can be executed to alter the inherent features of materials that are based on C, including electronic features and local and surface chemical characteristics [11]. Moreover, the NH_2_ groups that exist at the edge of functionalized amino groups and N-atom-doped GQDs have been reported to have the highest occupied molecular orbital due to a strong orbital interaction with the primary amine [14]. Therefore, the resonance between the delocalized *π* orbital and the primary amine molecular orbital narrows the orbital bandgap. Consequently, owing to their extraordinary edge and quantum confinement effects, the amino-N-GQDs were determined to have different chemical compositions to the GQDs, enhancing their photochemical, electrochemical, and electrocatalytic activities in optoelectronic and biomedical applications [2,6]. The increased ^1^O_2_ due to the amino-N-GQDs may, thus, have been the result of the triplet state yield of the amino-N-GQDs and more intersystem crossing [14]. These factors led to the superior antimicrobial activity of GQDs through the PDT mechanism. TEM was used to obtain images of MRSA treated with amino-N-GQDs exposed to a laser. After MRSA (Figure 6a) was incubated with amino-N-GQD–Ab_protein A_ for 3 h (Figure 6b), numerous materials were adsorbed on the bacterial surface [26]. Bacteria must assimilate nutrients and filter external ions to maintain and develop cell wall physiological functions. The uptake assay results revealed that a considerable amount of material was adsorbed on the bacterial surface [26,27], with a burst rate of approximately 72% within the first 3 h of incubation (Figure 5b). This rate saturated from 3 to 10 h. Therefore, the material was adsorbed on the bacterial surface and consequently constituted an external barrier. After 3 h of incubation, the material–Ab_protein A_-treated MRSA did not exhibit any exceptional morphology. However, the shape of the MRSA changed considerably after 4 days of incubation (Figure 6c), with a viability equal to nearly 62% (Figure 6e). This finding implies that the bacteria could not function normally and began to die. In particular, the material exhibited bacteriostatic or bactericidal abilities after 4 days of incubation. Moreover, after 3 h of additional incubation, the photoexcited material–Ab_protein A_-treated MRSA appeared substantially damaged, which resulted in an abnormal morphology (Figure 6d) corresponding to an elimination rate of almost 100% (Figure 6e).

**Figure 7:**
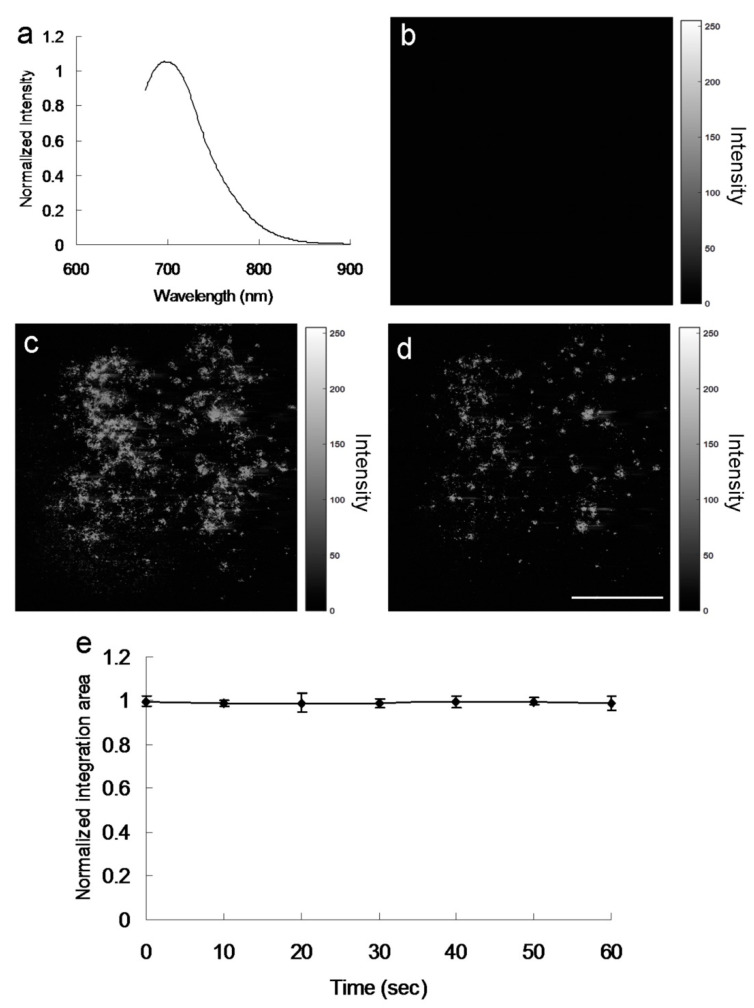
(**a**) Relative PL spectra of amino-N-GQDs exposed to one-photon excitation (Ex/Em: 670/696 nm). Gray-level luminescence images of (**b**) untreated MRSA and (**c**,**d**) amino-N-GQD–Ab_protein A_-treated MRSA and material-treated bacteria after photoexcitation for 60 s (0.07 W cm^−2^; Ex: 670 nm), respectively. On 3 h of additional incubation, the PDT action would be effectively processed. (**e**) Photostability assessment results. For emission wavelengths of 675–900 nm and as a function of exposure time (0−60 s), the relative intensity of the integrated area is almost constant, indicating high photostability. Delivered dose: OD_600_ = ~0.05 μg·mL^−1^ for bacteria and 0.5 μg·mL^−1^ for material–Ab_protein A_. Scale bar: 50 μm.

**Figure 8:**
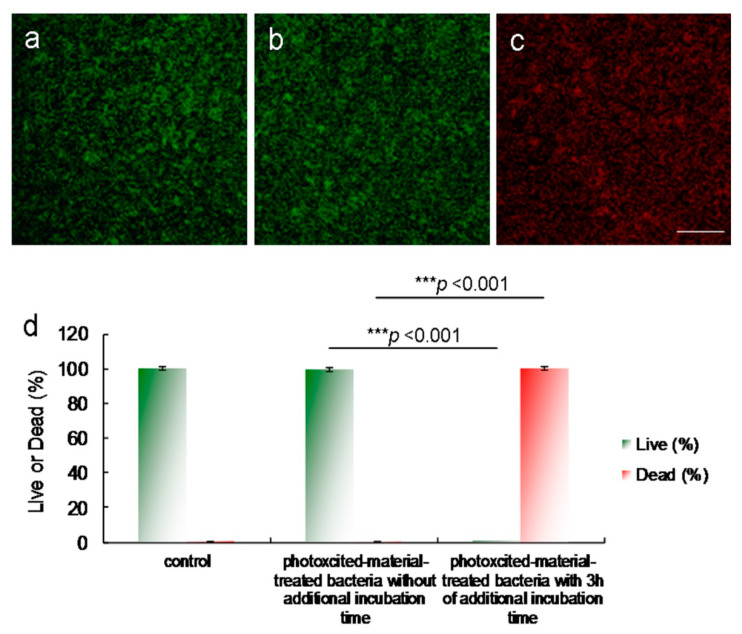
Images obtained after 60-s laser photoexcitation exposure (0.07 W·cm^−2^; Ex: 670 nm) of (**a**) bacteria alone with 3 h of additional incubation, and amino-N-GQD-Ab_protein A_-treated MRSA (**b**) without additional incubation, and (**c**) with 3 h of additional incubation. The LIVE/DEAD kit was used to stain bacteria before images were captured. (**d**) Viability (%) determination results. For the percentages of live and dead bacteria, *** *p* < 0.001. * *p* value was obtained using the Student’s *t* test (* *p* < 0.05, ** *p* < 0.01, *** *p* < 0.001). Delivered dose: OD_600_ = ~0.05 μg·mL^−1^ for bacteria and 0.5 μg·mL^−1^ for material–Ab_protein A_. Data are presented as mean ± SD (*n* = 6). Scale bar: 50 μm.

The surface groups of the amino-N-GQDs may contribute to intrinsic and defect state emissions that involve a PL mechanism [2,7,8,11]. Because of the amino functional groups on the amine-N-GQD surface, radiative electron–hole recombination was induced, which increased the intrinsic state emission [11,12]. The relative QY of amino-N-GQDs was estimated to be 0.34 (as reference, QY_ref_ = 0.28 for Cy5.5 in dimethyl sulfoxide [26,27]), whereas its absolute QY [28,29] was estimated to be 0.33. In contrast to the mentioned amino-N-GQDs, the GQDs were determined to have smaller relative and absolute QYs of 0.11 and 0.13, respectively. The PL spectrum of amino-N-GQDs strongly emitting NIR fluorescence at 696 nm, obtained using a fluorescence spectrometer (excitation: 670 nm), is displayed in Figure 7a. The laser-treated MRSA alone displayed no fluorescence emission (Figure 7b). When irradiation was applied, fluorescence from the materials on the bacterial surface was observed (Figure 7c). After 60 s of irradiation, the bacteria exhibited severe morphological damage. This behavior led to material desorption from the surface of the bacteria and caused an apparent decrease in fluorescence (Figure 7d). The amino-N-GQDs in this study also exhibited photostability, as indicated by their emission intensity after exposure. This photostability resulted in reduced photobleaching (Figure 7e) and high stability in physiological environments, even after 8 weeks (Table S6, which corresponds to Figures S8 and S9). MRSA viability was determined by fluorescence and quantification (Figure 8) [15]. After photoexcitation, the generated ROS required additional incubation to effectively process the PDT action. The green fluorescence indicative of living bacteria (Figure 8a) revealed that the bacteria exposed to laser treatment alone with 3 h of additional incubation were almost completely undamaged, which is consistent with the results presented in Figure 6a and Figure 7b. The photoexcited amino-N-GQD-Ab_LPS_-treated bacteria without additional incubation also showed nearly no damage (Figure 8b). After 3 h of additional incubation, the same panel revealed that the dead bacteria were distinguishable to an extent (represented by the red fluorescence in Figure 8c), which is consistent with the results presented in Figure 6d and Figure 7d. Bacterial viability was quantified for further antimicrobial testing. The viability tests indicated almost complete elimination of the material-treated bacteria (Figure 8d). Viability was also quantified using a CFU assay (Figure 3b and Figure S3b) to demonstrate the efficient antibacterial effect of the amino-N-GQDs in PDT. No other photochemical activity (i.e., photothermal effect) after photoexcitation was detected in this study.

## 3. Materials and Methods

### 3.1. Laser Exposure

A continuous-wave diode laser at 670 nm (Coherent, Inc., Santa Clara, CA, USA) was used for the laser irradiation experiments. Material–Ab (0.5 μg·mL^−1^) was treated with MRSA or *E. coli* (OD_600_ = ~0.05) and placed in a 96-well cell culture plate (Catalog number 174925, Thermo Fisher Scientific, Waltham, MA, USA). Thereafter, the material–Ab-treated-MRSA or -*E. coli* was incubated at 37 °C in the dark. The laser was focused and irradiated on the sample. Relevant experiments were then conducted.

### 3.2. Singlet Oxygen Quantum Yield (Φ_Δ_) Measurement

Φ_Δ_ was obtained from a previous study. Φ_Δ_ measurements were conducted in D_2_O at 355 nm using *meso*-tetra (4-sulfonatophenyl) porphine dihydrochloride (CAS number 139050-15-0, Sigma Aldrich Co., St. Louis, MO, USA) as a reference (Φ_Δ_ = 0.64) [18].

### 3.3. PL QY Measurement

The PL *QY* of the contrast agent is usually the ratio of the emitted photons to the absorbed photons, and is calculated as follows:

(1) $$QY=QY_{ref}\left(\eta^{2}/\eta_{ref}^{2}\right)\left(I/A\right)\left(A_{ref}/I_{ref}\right)$$

where *QY_ref_* is the *QY* of Cy5.5 (CAS number 2260669-71-2, Thermo Fisher Scientific, Waltham, MA, USA) dissolved in dimethyl sulfoxide (DMSO) as a reference, *η* is the refractive index of ddH_2_O = 1.3333 (*η*_ref_ of DMSO = 1.479), *A* is the absorbance at the excitation wavelength, and *I* is the integrated PL intensity.

All Materials and Methods for this article can be found in the Supplementary Materials.

## 4. Conclusions

GQDs have been utilized in numerous research fields. However, their use as PSs to directly generate ROS has not received much attention. In this study, we fabricated amino-N-GQDs. The amino-N-GQDs could generate ROS and were effectively used in PDT to eliminate the Gram-positive MDR bacteria, MRSA, at low energy levels within an extremely short photoexcitation period. Amino-N-GQDs have properties, such as high absorption efficiency, strong luminescence, and high stability, making them promising contrast agents for use in biological specimens. Consequently, amino-N-GQDs can be used to perform dual-modality PDT and bioimaging, providing an alternative means for efficiently managing malignant and MDR species.

## Figures and Tables

**Table 1:** MRSA viability (%), determined from the viable count of material–Ab_protein A_-treated MRSA obtained using the CFU assay under a short 670-nm laser excitation. Data are presented as mean ± SD (*n* = 6).

**0.5 μg·mL^−1^**	**GQD**		**N-GQD**		**amino-GQD**		**amino-N-GQD**	
	**3 h-** **Incubation**	**6 h-** **Incubation**	**3 h-** **Incubation**	**6 h-** **Incubation**	**3 h-** **Incubation**	**6 h-** **Incubation**	**3 h-** **Incubation**	**6 h-** **Incubation**
0-s photoexcitation	99 ± 1%	99 ± 2%	100 ± 1%	99 ± 1%	98 ± 2%	99 ± 2%	100 ± 1%	99 ± 1%
30-s photoexcitation	75 ± 4%	76 ± 3%	66 ± 2%	68 ± 3%	67 ± 4%	70 ± 2%	36 ± 3%	35 ± 2%
60-s photoexcitation	37 ± 3%	35 ± 3%	24 ± 2%	23 ± 2%	24 ± 3%	26 ± 2%	0%	0%
**1.0 μg·mL^−1^**	**GQD**		**N-GQD**		**amino-GQD**		**amino-N-GGD**	
	**3 h-** **Incubation**	**6 h-** **Incubation**	**3 h-** **Incubation**	**6 h-** **Incubation**	**3 h-** **Incubation**	**6 h-** **Incubation**	**3 h-** **Incubation**	**6 h-** **Incubation**
0-s photoexcitation	99 ± 2%	100 ± 1%	98 ± 1%	99 ± 2%	99 ± 1%	98 ± 2%	99 ± 2%	100 ± 1%
30-s photoexcitation	43 ± 3%	44 ± 3%	35 ± 3%	35 ± 4%	34 ± 4%	35 ± 5%	17 ± 2%	16 ± 2%
60-s photoexcitation	19 ± 2%	20 ± 3%	10 ± 1%	11 ± 1%	10 ± 2%	12 ± 3%	0%	0%

**Table 2:** Amount of ROS generated by conducting photoexcitation (0.07 W cm^−2^; Ex: 670 nm) and by using materials–Ab-treated bacteria was maintained in the dark and monitored. Delivered dose: OD_600_ = ~0.05 for MRSA and 0.5 μg·mL^−1^ for material. Data are presented as mean ± SD (*n* = 6) [6,14,22].

	**Negative** **Control ^ac^**	**ROS** **Neutralization ^abc^**	**^1^** **O_2_ (by SOSG) ^c^**		**ROS** **Neutralization ^bc^**	**GQD**	**ROS** **Neutralization ^bc^**
			**Positive** **Control ^cd^**	**amino-N-** **GQD**			
30-s photoexcitation 60-s photoexcitation	239 ± 10 241 ± 11	241 ± 12 242 ± 9	1956 ± 81 2689 ± 137	1024 ± 39 1735 ± 55	241 ± 12 241 ± 11	505 ± 23 878 ± 30	240 ± 9 242 ± 10
	**Negative** **control ^ac^**	**ROS** **neutralization ^abe^**	**^1^** **O_2_ (by *t*-MVP) ^e^**		**ROS** **neutralization ^be^**	**GQD**	**ROS** **neutralization ^be^**
			**Positive** **control ^cd^**	**amino-N-** **GQD**			
30-s photoexcitation 60-s photoexcitation	323 ± 19 326 ± 22	322 ± 19 327 ± 21	6043 ± 141 8584 ± 175	4107 ± 102 5362 ± 133	322 ± 21 328 ± 20	2149 ± 57 3006 ± 82	323 ± 20 327 ± 21
	**Negative** **control ^ac^**	**ROS** **neutralization ^abf^**	**O_2_^−^ (by XTT) ^f^**		**ROS** **neutralization ^bf^**	**GQD**	**ROS** **neutralization ^bf^**
			**Positive** **control ^cd^**	**amino-N-** **GQD**			
30-s photoexcitation 60-s photoexcitation	0 0	0 0	1.13 ± 0.08 1.99 ± 0.14	0.85 ± 0.07 1.40 ± 0.11	0.02 ± 0.01 0.03 ± 0.02	0.55 ± 0.06 0.87 ± 0.07	0.02 ± 0.01 0.03 ± 0.01
	**Negative** **control ^ac^**	**ROS** **neutralization ^abg^**	**O_2_** **^−^ (by GSH) ^g^**		**ROS** **neutralization ^bg^**	**GQD**	**ROS** **neutralization ^bg^**
			**Positive** **control ^cd^**	**amino-N-** **GQD**			
30-s photoexcitation 60-s photoexcitation	0 0	0 0	77.3 ± 2.8% 99.6 ± 4.5%	61.8 ± 2.2% 83.6 ± 3.1%	0.3 ± 0.1% 0.2 ± 0.1%	37.3± 1.5% 52.0 ± 2.2%	0.3 ± 0.2% 0.3 ± 0.1%

^a^ Negative control: only treat using reagent and laser radiation without using any material (0 μg·mL^−1^). ^b^ ROS neutralization includes nanomaterial treatment, laser irradiation, and 30 ppm of antioxidant *α*-Tocopherol/methyl linoleate. ^c^ The singlet oxygen sensor green (SOSG) reagent (Ex/Em: 488/525 nm) has a specific reactivity to generate fluorescence that is recorded using a PL spectrometer. ^d^ Positive control: treatment of 50 μM of *tert*-butyl hydroperoxide and laser irradiation. ^e^ trans-1-(2′-methoxyvinyl)pyrene (*t*-MVP) (Ex/Em: 352/465 nm) can react with ^1^O_2_, and form a dioxetane intermediate that generates fluorescence upon decomposition to 1-pyrenecarboxaldehyde. The process is monitored using a PL spectrometer. ^f^ 2,3-bis (2-methoxy-4-nitro-5-sulfophenyl)-2H-tetrazolium-5-carboxanilide (XTT) can interact with O_2_^−^ and produce XTT-formazan that generates strong absorption (wavelength: 470 nm). ^g^ *γ*-l-glutamyl-l-cysteinyl-glycine (GSH) containing a thiol tripeptide can prevent damages to cellular or bacterial components caused by stress of oxidation. The thiol group from GSH can be oxidized to the disulfide bond, thus converting GSH to glutathione disulfide. GSH oxidation was used to determine the generated O_2_**^−^**. Loss of GSH (%) = (difference between of the absorbance of the sample and negative control/absorbance of negative control) × 100%.

## Data Availability

Not applicable.

## Supplementary Materials

The following are available online at https://www.mdpi.com/article/10.3390/ijms22189695/s1.
