# Supplementary material for: Nitrogen Functionalities of Amino-Functionalized Nitrogen-Doped Graphene Quantum Dots for Highly Efficient Enhancement of Antimicrobial Therapy to Eliminate Methicillin-Resistant Staphylococcus aureus and Utilization as a Contrast Agent
Source: Int J Mol Sci. 2021 Sep 7;22(18):9695. doi: 10.3390/ijms22189695 (PMC8468865; doi:10.3390/ijms22189695)
Supplement: Supplementary file 1 [file ijms-22-09695-s001.zip › ijms-1313160-supplementary.pdf]

Supplementary Materials

Nitrogen functionalities of amino-functionalized nitrogen-doped graphene quantum dots for highly efficient enhancement of antimicrobial therapy to eliminate methicillin-resistant *Staphylococcus aureus* and utilization as a contrast agent

Wen-Shuo Kuo[1,2,3,4,†], *Ping-Ching Wu*[5,6,7,8,†], *Chi-Yao Hung*[9,†], *Chia-Yuan Chang*[10], *Jiu-Yao Wang*[3,4], *Pei-Chi Chen*[3,4], *Miao-Hsi Hsieh*[3,4], *Sheng-Han Lin*[11*], *Chan-Chi Chang*[12*], *and Yen-Sung Lin*[13,14*]

[1]School of Chemistry and Materials Science, Nanjing University of Information Science and Technology, Nanjing 210044, Jiangsu, China

[2]State Key Laboratory for Chemistry and Molecular Engineering of Medicinal Resources, Guangxi Normal University, Guilin 541004, China

[3]Allergy & Clinical Immunology Research Center, National Cheng Kung University Hospital, College of Medicine, National Cheng Kung University, Tainan 701, Taiwan (R.O.C.)

[4]Allergy Immunology and Microbiome Center, China Medical University Children's Hospital, China Medical University, Taichung 404, Taiwan (R.O.C.)

[5]Department of Biomedical Engineering, National Cheng Kung University, Tainan 701, Taiwan (R.O.C.)

[6]Institute of Oral Medicine and Department of Stomatology, National Cheng Kung University Hospital, College of Medicine, National Cheng Kung University Tainan 701, Taiwan (R.O.C.)

[7]Center of Applied Nanomedicine, National Cheng Kung University, Tainan, Taiwan (R.O.C.)

[8]Medical Device Innovation Center, Taiwan Innovation Center of Medical Devices and Technology, National Cheng Kung University Hospital, National Cheng Kung University, Tainan 701, Taiwan (R.O.C.)

[9]Department of Physical Medicine and Rehabilitation, An Nan Hospital, China Medical University, Tainan 709, Taiwan (R.O.C.)

[10]Department of Mechanical Engineering, National Cheng Kung University, Tainan 701, Taiwan (R.O.C.)

[11]Department of Anesthesiology, E-Da Hospital, Kaohsiung 824, Taiwan (R.O.C.)

[12]Department of Otolaryngology, National Cheng Kung University Hospital, College of Medicine, National Cheng Kung University, Tainan 701, Taiwan (R.O.C.)

[13]Division of Pulmonary and Critical Care Medicine, An Nan Hospital, China Medical University, Tainan, 709, Taiwan (R.O.C.)

[14]Department of Nursing, Chung Hwa University of Medical Technology, Tainan 717, Taiwan (R.O.C.)

[†]The authors contributed equally to this work.

*To whom correspondence should be addressed. E-mail: ed111667@edah.org.tw (S.-H.L.); 109a0015@gs.ncku.edu.tw (C.-C.C.); chestlin@gmail.com (Y.-S.L.)

Materials and Methods

Materials [5,15,17,20]

GQDs: Graphene oxide was prepared using a natural graphite powder (Bay carbon Inc., Bay City, MI, USA) through a modified Hummers' method. Graphite (8.5 M) and $NaNO_3$ (0.6 M) (CAS number 7631-99-4, Merck & Co., Kenilworth, NJ, USA) were mixed with H_2SO_4 (CAS number 7664-93-9, FUJIFILM Wako Chemicals USA Inc., Richmond, VA, USA). $KMnO_4$ (2.0 M, CAS number 7722-64-7, FUJIFILM Wako Chemicals USA Inc., Richmond, VA, USA) was slowly added with continual stirring at 35 °C (PC400, Corning, New York, NY, USA) overnight. Subsequently, deionized water (ddH_2O) was gradually added and continually stirred. H_2O_2 (CAS number 7722-84-1, Sigma Aldrich Co., St Louis, MO, USA) was added to terminate the reaction. Washing and centrifugation with ddH_2O were performed several times, and the graphene oxide was collected. The as-prepared graphene oxide was placed in a tube furnace (Tainan, Taiwan) and heated to 400–600 °C in the presence of argon for 4–6 h; it was subsequently introduced to concentrated HNO_3 (16.0 M, CAS number 7697-37-2, Sigma Aldrich Co., St Louis, MO, USA) and stirred for 2 d. The mixture was placed in a water bath sonicator (Soner 220H, Rocker Scientific Co. Ltd., New Taipei City, Taiwan) for 2 d and maintained the temperature of water was approximately 45 °C; subsequently placed in an oven at 160 °C (DOV-40N, Yotec Instruments Co. Ltd., Hsinchu City, Taiwan) for 1 d to vaporize all the liquid. Washing and centrifugation (approximately 847200 RCF, > 10 min; Optima TLX Ultracentrifuge, Beckman Coulter Inc., Brea, CA, USA) with ddH_2O were conducted several times. The supernatant was filtered through a 0.22 μm microporous membrane (Catalog number Z290807-100EA, Nylon filter membrane, Merck & Co., Kenilworth, NJ, USA). The pH (7.4; HI2002, Hanna Co. Ltd., Thane, Maharashtra, India) of the resulting black suspension was tuned with NaOH (CAS number 1310-73-2, Sigma Aldrich Co., St Louis, MO, USA) to obtain the GQDs specimens (**Scheme S1a**).

N-GQDs: Graphene oxide was prepared from a natural graphite powder using a modified Hummers' method. Graphite (8.5 M) and $NaNO_3$ (0.6 M) were mixed with H_2SO_4. $KMnO_4$ was slowly added with continual stirring at 35 °C overnight. Subsequently, deionized water (ddH_2O) was gradually added and continually stirred. H_2O_2 was added to terminate the reaction. Washing and centrifugation with ddH_2O were performed several times, and the graphene oxide was collected. The as-prepared graphene oxide was placed in a tube furnace and heated to 400–600 °C in the presence of ammonia (CAS number 7664-41-7, Sigma Aldrich Co., St Louis, MO, USA) for 4–6 h; it was subsequently introduced to concentrated HNO_3 (16.0 M) and stirred for 2 d. The mixture was placed in a water bath sonicator for 2 d and maintained the temperature of water was approximately 45 °C; subsequently placed in an oven at 160 °C for 1 d to vaporize all the liquid. Washing and centrifugation (approximately 847200 RCF, > 10 min) with ddH_2O were conducted several times. The supernatant was filtered through a 0.22 μm microporous membrane. The pH (7.4) of the resulting black suspension was tuned with NaOH to obtain the N-GQDs specimens.

Amino-GQDs: The as-prepared GQDs were mixed with ammonia, stored in a Teflon-lined stainless steel autoclave (Tainan, Taiwan), and reacted at 180 °C for 5 h. The resulting mixture was washed with ddH_2O, centrifuged several times, and subsequently dried in an oven at 50 °C overnight to obtain the amino-GQDs specimens.

Amino-N-GQDs: The as-prepared N-GQDs were mixed with ammonia, stored in a Teflon-lined stainless steel autoclave, and reacted at 180 °C for 5 h. The resulting mixture was washed with ddH_2O, centrifuged several times, and subsequently dried in an oven at 50 °C overnight to obtain the amino-N-GQDs specimens (**Scheme S1b**).

A 0.5 mg mL^{-1} or 5 mg mL^{-1} stock solution of GQDs, N-GQDs, amino-GQDs or amino-N-GQDs was prepared for the following experiments.

Characterization

Materials were subject to transmission electron microscopy (TEM; JEOL 2100F and JEOL 3010, Akishima, Tokyo, Japan) observation. Fourier-transform infrared (FTIR) spectroscopy, ultraviolet-visible

(UV-vis), X-ray diffraction (XRD) and zeta potential spectra / dynamic light scattering (DLS) of samples were recorded by the spectrometers, respectively: RX1, PerkinElmer, Waltham, MA, USA; U-4100 Hitachi, Chiyoda-ku, Tokyo; Bruker AXS Gmbh, D2 Phaser, Billerica, MA, USA; and Malvern Nano-ZS90, Worcestershire, West Midlands, UK, respectively. Raman spectroscopy (DXR, Thermo Fisher Scientific, Waltham, MA, USA) was used to examine the crystallinity of samples with 532 nm laser. X-ray photoelectron spectroscopy (XPS; PHI 5000, VersaProbe, Chanhassen, MN, USA**)** was employed to examine the surface chemistry of the materials, the O(1s) / C(1s) and N(1s) / C(1s) atomic ratios of materials. The PL signal was recorded by the spectrophotometer (F-7000, Hitachi, Chiyoda-ku, Tokyo, Japan). Delivered dose: from 0.5 µg mL^{-1} to 5 mg mL^{-1} material.

Bacterial culture

For methicillin-resistant *Staphylococcus aureus* (MRSA, ATCC 27659; purchased from Food Industry Research and Development Institute, Hsinchu City, Taiwan):

Brain Heart Infusion (BHI) agar plates (Catalog number 08-757-100D, Falcon Bacteriological Petri Dishes with Lid; Corning, New York, NY, USA): Add 52 g of BHI agar (Catalog number 241830, DIFCO, BD, Franklin Lakes, NJ, USA) to ddH_2O in a 2L flask. Add a stir bar to the flask and then place the flask on a stir plate to mix. Bring volume up to 1L using ddH_2O. Loosely cover the top of the flask with aluminum foil and then autoclave (STA-400, St. Francis Medical Equipment Co. Ltd., Taipei, Taiwan) the media on liquid cycle. Once sterilized, place the flask on a stir plate until the media has cooled to 65 °C. Allow agar to solidify at room temperature for at least 1 d then store the plates, top down, in plastic bags at 4 °C (Bioway Co. Ltd., Tainan, Taiwan).

BHI broth: Add 37 g of BHI (Catalog number 237500, BACTO, BD, Franklin Lakes, NJ, USA) to ddH_2O in a 2L flask. Add a stir bar to the bottle and then place the bottle on a stir plate to mix. Bring volume up to 1L using ddH_2O. Once the powder is completely dissolved, remove the stir bar, loosely cap the bottle and autoclave the media on liquid cycle. Once sterilized, the broth may be stored at room temperature or 4 °C.

Reviving freeze-dried MRSA: For freeze dried cultures, using a single tube (Catalog number 07-000-215, Fisher Scientific, Hampton, NH, USA) of the BHI broth (5-6 mL) and withdraw 0.5-1.0 mL with pipette (SKU F123602, Gilson Inc., Middleton, NI, USA). Use this to rehydrate the entire pipette and transfer the entire suspension back into the broth tube and mix well. The last few drops of this suspension may also be transferred to an agar slant. The anaerobic cultures must be rehydrated only in an anaerobic environment; the viability of the bacteria decreases rapidly if the vial is rehydrated in an oxygenic environment. Incubate cultures at 37 °C (WY-304, Wood Yellow Co. Ltd., Taipei, Taiwan) and most freeze-dried cultures will grow out in a few days.

Thawing frozen MRSA: For frozen cultures, thaw MRSA using gentle agitation in a water bath (BC-2D, Taiwan Hipoint Co. Ltd., Kaohsiung, Taiwan) that is set to 25-30°C. Thawing will be rapid; ~2 mins or until all ice crystals have melted. For anaerobic cultures, thaw the vial only in an anaerobic environment; the viability of the cell decrease rapidly if the vial is thawed in an oxygenic environment. Once the vial is thawed, decontaminate the outer surface using 70% ethanol (CAS number 64-17-5, Sigma Aldrich Co., St Louis, MO, USA). The entire contents of the vial can then be aseptically transferred to a test tube containing 5-6 mL of BHI broth. Incubate the culture at 37 °C. Additional test tubes can be inoculated by transferring 0.5 mL of the culture of the primary culture to additional secondary cultures.

Growth of MRSA on BHI agar plates: Using a sterile applicator streak out a small amount of MRSA from the frozen stock onto a small section of a BHI agar plate using aseptic technique. With a new sterile applicator, streak out a quadrant MRSA by passing through the initial quadrant several times. Repeat this process 1-2 more times, passing a new applicator through most recently streaked quadrant. Incubate the plates for 16-24 h at 37 °C.

Growth of MRSA in BHI broth: MRSA grows rapidly in broth culture at 37 °C with aeration. As such, overnight cultures of MRSA are typically started at the end of the day just prior to leaving the LAB using either an isolated colony from an agar plate or a frozen stock. Using septic technique and transfer a single colony of MRSA from the streak plate into the aliquoted broth by tilting the culture tube and rubbing the

inoculating loop against the side of the tube at the liquid-air interface. Grow cultures overnight (~16-18 h) at 37 °C with shaking (Catalog number 757, Amerex Instruments, Inc., Concord, CA, USA).

Preparation of MRSA frozen stocks: Long-term storage of MRSA should occur at -80 °C (MDF-U76VA-PA, LABRepCo, Horsham, PA, USA) to prevent accumulation of mutations. To prepare a frozen stock of MRSA add an aliquot (850 μL) of an overnight culture to sterile dimethyl sulfoxide (DMSO, 150 μL, CAS number 67-68-5, Sigma Aldrich Co., St Louis, MO, USA) to one sterile cryotube (Catalog number 479-6843, Avantor, Radnor, PA, USA) using aseptic technique and vortex / or invert briefly to mix. The addition of DMSO prevents complete freezing of the bacteria and thus limits bacterial damage as result of the transition to -80 °C.

For *Escherichia coli* (*E. coli*, ATCC 25922; obtained from our own laboratory):

LB agar plates (Catalog number 08-757-100D, Falcon Bacteriological Petri Dishes with Lid; Corning, New York, NY, USA): Add 10 g of tryptone (Catalog number 91079-40-2, Sigma Aldrich Co., St Louis, MO, USA), 5 g of yeast extract (Catalog number 8013-01-2, Sigma Aldrich Co., St Louis, MO, USA), 8 g of sodium chloride (Catalog number 7647-14-5, Sigma Aldrich Co., St Louis, MO, USA) and 15 g of agar (Catalog number L3027, Sigma Aldrich Co., St Louis, MO, USA) to ddH_2O in a 2L flask. Add a stir bar to the flask, place the flask on a stir plate to mix and tune the pH to 7.5. Bring volume up to 1L using ddH_2O. Loosely cover the top of the flask with aluminum foil and then autoclave (STA-400, St. Francis Medical Equipment Co. Ltd., Taipei, Taiwan) the media on liquid cycle. Once sterilized, place the flask on a stir plate until the media has cooled to 65 °C. Allow agar to solidify at room temperature for at least 1 d then store the plates, top down, in plastic bags at 4 °C (Bioway Co. Ltd., Tainan, Taiwan).

LB broth: Add 10 g of tryptone (Catalog number 91079-40-2, Sigma Aldrich Co., St Louis, MO, USA), 5 g of yeast extract (Catalog number 8013-01-2, Sigma Aldrich Co., St Louis, MO, USA) and 8 g of sodium chloride (Catalog number 7647-14-5, Sigma Aldrich Co., St Louis, MO, USA) to ddH_2O in a 2L flask. Add a stir bar to the bottle and then place the bottle on a stir plate to mix. Bring volume up to 1L using ddH_2O. Once the powder is completely dissolved, remove the stir bar, loosely cap the bottle and autoclave the media on liquid cycle. Once sterilized, the broth may be stored at room temperature or 4 °C.

Reviving freeze-dried *E. coli*: For freeze dried cultures, using a single tube (Catalog number 07-000-215, Fisher Scientific, Hampton, NH, USA) of the LB broth (5-6 mL) and withdraw 0.5-1.0 mL with pipette (SKU F123602, Gilson Inc., Middleton, NI, USA). Use this to rehydrate the entire pipette and transfer the entire suspension back into the broth tube and mix well. The last few drops of this suspension may also be transferred to an agar slant. The anaerobic cultures must be rehydrated only in an anaerobic environment; the viability of the bacteria decreases rapidly if the vial is rehydrated in an oxygenic environment. Incubate cultures at 37 °C (WY-304, Wood Yellow Co. Ltd., Taipei, Taiwan) and most freeze-dried cultures will grow out in a few days.

Thawing frozen *E. coli*: For frozen cultures, thaw *E. coli* using gentle agitation in a water bath (BC-2D, Taiwan Hipoint Co. Ltd., Kaohsiung, Taiwan) that is set to 25-30°C. Thawing will be rapid; ~2 mins or until all ice crystals have melted. For anaerobic cultures, thaw the vial only in an anaerobic environment; the viability of the cell decrease rapidly if the vial is thawed in an oxygenic environment. Once the vial is thawed, decontaminate the outer surface using 70% ethanol (CAS number 64-17-5, Sigma Aldrich Co., St Louis, MO, USA). The entire contents of the vial can then be aseptically transferred to a test tube containing 5-6 mL of LB broth. Incubate the culture at 37 °C. Additional test tubes can be inoculated by transferring 0.5 mL of the culture of the primary culture to additional secondary cultures.

Growth of *E. coli* on LB agar plates: Using a sterile applicator streak out a small amount of *E. coli* from the frozen stock onto a small section of a LB agar plate using aseptic technique. With a new sterile applicator,

streak out a quadrant *E. coli* by passing through the initial quadrant several times. Repeat this process 1-2 more times, passing a new applicator through most recently streaked quadrant. Incubate the plates for 16-24 h at 37 °C.

Growth of *E. coli* in LB broth: *E. coli* grows rapidly in broth culture at 37 °C with aeration. As such, overnight cultures of *E. coli* are typically started at the end of the day just prior to leaving the LAB using either an isolated colony from an agar plate or a frozen stock. Using septic technique and transfer a single colony of *E. coli* from the streak plate into the aliquoted broth by tilting the culture tube and rubbing the inoculating loop against the side of the tube at the liquid-air interface. Grow cultures overnight (~16-18 h) at 37 °C with shaking (Catalog number 757, Amerex Instruments, Inc., Concord, CA, USA).

Preparation of *E. coli* frozen stocks: Long-term storage of *E. coli* should occur at -80 °C (MDF-U76VA-PA, LABRepCo, Horsham, PA, USA) to prevent accumulation of mutations. To prepare a frozen stock of *E. coli* add an aliquot (850 μL) of an overnight culture to sterile dimethyl sulfoxide (DMSO, 150 μL, CAS number 67-68-5, Sigma Aldrich Co., St Louis, MO, USA) to one sterile cryotube (Catalog number 479-6843, Avantor, Radnor, PA, USA) using aseptic technique and vortex / or invert briefly to mix. The addition of DMSO prevents complete freezing of the bacteria and thus limits bacterial damage as result of the transition to -80 °C.

Coating antibody

The absorbance of a certain quantity of $Ab_{protein\ A}$ or Ab_{LPS} (abcam, Cambridge, UK) was recorded via UV-vis spectroscopy (Abs: approximately from 200 nm to 240 nm). By electrostatic interaction, the materials were mixed with the same quantity antibody for 30 min during incubation at 4 °C in the dark and centrifuged (approximately 847200 RCF, > 10 min) to remove excess antibody; the material-$Ab_{protein\ A}$ or -Ab_{LPS} was then prepared. Conversely, the supernatant was retained and its absorbance was measured. The difference between the absorbance of the collected supernatant and the original antibody was estimated. Consequently, the quantity of the antibody absorbed on the materials was calculated using Lambert-Beer's law [$A= \varepsilon bC$, where A= absorbance, ε= molar extinction coefficient, b= path length (1 cm), and C= concentration]. In the working solution of 1×PBS buffer, approximately 0.12 μg of $Ab_{protein\ A}$ was absorbed on 1 μg of amino-N-GQD. This implies that the absorption efficiency was approximately 12.0% (zeta potential of amino-N-GQD-$Ab_{protein\ A}$: 13.7 mV / in 1×PBS buffer solution) and 10.9% (13.2 mV of zeta potential / in MRSA BHI broth), respectively. For GQD-$Ab_{protein\ A}$, the efficiency of absorption was approximately 10.7% (12.5 mV of zeta potential / in 1×PBS buffer solution) and 10.1% (11.4 mV of zeta potential / in MRSA BHI broth), respectively. On the other hand, the absorption efficiency was approximately 13.1% (zeta potential of amino-N-GQD-$Ab_{protein\ A}$: 14.5 mV / in 1×PBS buffer solution) and 12.2% (13.9 mV of zeta potential / in *E. coli* LB broth), respectively. For GQD-$Ab_{protein\ A}$, the efficiency of absorption was approximately 12.5% (13.0 mV of zeta potential / in 1×PBS buffer solution) and 11.0% (12.6 mV of zeta potential / in MRSA BHI broth), respectively. As there is not much difference between the zeta potential of material-Ab in the 1×PBS buffer solution, MRSA BHI broth and *E. coli* LB broth, it meant that the biomolecules would be absorbed on neither material and Ab nor material-Ab. In other words, the interaction between Ab, material-Ab and bacteria would not be influenced by biomolecules in the MRSA or *E. coli* culture media, thus leading to no subsequently effect in the specific binding between them. Moreover, the positively charged material-Ab was favorable for absorbance or internalization by the negatively charged bacterial surface (**Schemes S1c-d**). The aforementioned results have proven the successful absorption of Ab on the surface of materials (**Table S7**).

Laser exposure

A continuous-wave diode laser at 670 nm (Coherent, Inc., Santa Clara, CA, USA) was selected for the laser irradiation experiments. Material-Ab (0.5 μg mL^{-1}) was treated with MRSA or *E. coli* (OD_{600} ~0.05) and put in the 96-well cell culture plate (Catalog number 174925, Thermo Fisher Scientific, Waltham, MA, USA). Then, the material-Ab-treated-MRSA or -*E. coli* was subjected to incubate at 37 °C in darkness. The laser was focused and irradiated on the sample. The relevant experiments were then followed up and conducted.

ROS detection [5,15,17,20]

Singlet oxygen ($^1O_2$): (a) Material-Ab (0.5 μg mL^{-1}) was treated with MRSA or *E. coli* (OD_{600} ~0.05). Then, the material-Ab-treated-MRSA or -*E. coli* was subjected to incubate at 37 °C in darkness. Subsequently, the mixture was exposed to a 60-s 670-nm laser (output power: 0.07 W cm^{-2}) and finally mixed with a Singlet Oxygen Sensor Green (SOSG) reagent (1 μM, Catalog number S36002, Thermo Fisher Scientific, Waltham, MA, USA) (Ex/Em: 488/525 nm). A fluorescence spectrometer was employed for measurements. For ROS neutralization, the mixture was mixed with 30 ppm of antioxidant *α*-tocopherol (CAS number 10191-41-0, Sigma Aldrich Co., St Louis, MO, USA.) / methyl linoleate (CAS number 301-00-8, Sigma Aldrich Co., St Louis, MO, USA.) in darkness, and then, the mixture was exposed to a 60-s 670-nm laser (output power: 0.07 W cm^{-2}) with the same treatment. (b) Material-Ab (0.5 μg mL^{-1}) was treated with MRSA or *E. coli* (OD_{600} ~0.05). Then, the material-Ab-treated-MRSA or -*E. coli* was subjected to incubate at 37 °C in darkness. Subsequently, the mixture was exposed to a 60-s 670-nm laser (output power: 0.07 W cm^{-2}) and finally mixed with 10 μM trans-1-(2′-methoxyvinyl)pyrene (*t*-MVP, Catalog number M7913, Thermo Fisher Scientific, Waltham, MA, USA) / 0.10 M sodium dodecyl sulfate (SDS, CAS number 151-21-3, Sigma Aldrich Co., St Louis, MO, USA) (Ex / Em: 352 nm / 465 nm). For ROS neutralization, the mixture was mixed with 30 ppm of antioxidant *α*-tocopherol / methyl linoleate in darkness. The reaction between *t*-MVP and $^1O_2$ yields a dioxetane intermediate that fluoresces while it decomposes into 1-pyrenecarboxaldehyde. This highly selective fluorescent probe does not react with other activated oxygen species such as hydroxyl radicals, superoxide, and hydrogen peroxide. A fluorescence spectrometer was employed for measurements. ROS neutralization was conducted with the same treatment that is described previously.

Superoxide radical anion ($O_2^{\cdot-}$): (a) Material-Ab (0.5 μg mL^{-1}) was treated with MRSA or *E. coli* (OD_{600} ~0.05). Then, the material-Ab-treated-MRSA or -*E. coli* was subjected to incubate at 37 °C in darkness. Subsequently, the mixture was exposed to a 60-s 670-nm laser (output power: 0.07 W cm^{-2}) and finally mixed with 2,3-bis (2-methoxy-4-nitro-5-sulfophenyl)-2H-tetrazolium-5-carboxanilide (XTT, 0.45 mM, CAS number 111072-31-2, Sigma Aldrich Co., St Louis, MO, USA). The purpose of this material was that it interacted with $O_2^{\cdot-}$ and produced XTT-formazan, thus resulting in a strong absorption (wavelength: 470 nm). A UV-vis spectrometer was employed to monitor this absorption. For ROS neutralization, the mixture was mixed with 30 ppm of antioxidant *α*-tocopherol/methyl linoleate in darkness and exposed to a 60-s 670-nm laser (output power: 0.07 W cm^{-2}) with the same treatment. (b) Material-Ab (0.5 μg mL^{-1}) was treated with MRSA or *E. coli* (OD_{600} ~0.05). Then, the material-Ab-treated-MRSA or -*E. coli* was subjected to incubate at 37 °C in darkness. Subsequently, the mixture was exposed to a 60-s 670-nm laser (output power: 0.07 W cm^{-2}) and finally mixed with 50-mM bicarbonate buffer (pH 8.60, Catalog number 88975, Merck & Co., Kenilworth, NJ, USA) and glutathione (*γ*-L-glutamyl-L-cysteinyl-glycine, GSH, CAS number 70-18-8, Sigma Aldrich Co., St Louis, MO, USA) / 0.80 mM bicarbonate buffer (Ellman's assay for $O_2^{\cdot-}$ detection). Subsequently, the following experiments were conducted based on the procedure in a previous study. Loss of GSH (%) was calculated as the difference between the absorbance of the sample and negative control divided by the absorbance of the negative control. The signal of the generated $O_2^{\cdot-}$ was obtained as described in the previous calculation. Data are presented as mean ± SD (*n*= 6).

Singlet oxygen quantum yield (Φ_Δ) measurement [28]

From previous study, Φ_Δ can be obtained. Φ_Δ measurements were conducted in D_2O at 355 nm by using *meso*-tetra(4-sulfonatophenyl)porphine dihydrochloride (CAS number 139050-15-0, Sigma Aldrich Co., St Louis, MO, USA) as a reference (Φ_Δ= 0.64).

PL quantum yield (QY) measurement

The PL QY of the contrast agent is usually the ratio of the emitted photons to the absorbed photons and is given as follows:

$$QY = QY_{ref}\left(\eta^2/\eta_{ref}^2\right)(I/A)\left(A_{ref}/I_{ref}\right) \tag{S1}$$

, where QY_{ref} is the QY of Cy5.5 (CAS number 2260669-71-2, Thermo Fisher Scientific, Waltham, MA, USA) dissolved in DMSO as a reference, η is the refractive index of ddH_2O= 1.3333 (η_{ref} of DMSO= 1.479), A is the absorbance at an excitation wavelength and I is the integrated PL intensity.

Biocompatibility assay of MRSA or *E. coli with the colony forming unit (CFU) counting method* [15,17,20]

MRSA or *E. coli* (OD_{600}: ~0.05) were added with materials-Ab (0-1.5 μg mL^{-1}) by incubating at 37 °C in darkness. After incubation, the mixture was centrifuged (approximately 1500 RCF for 20 min, KUBOTA-2420, Kaohsiung, Taiwan). In this step, the material was absorbed by bacteria or the material-treated bacteria can be was centrifuged and pulled down as pellets. Before plating, dilution should be carried out in 10 folds steps using the maximum recovery dilution medium (*Note: In preparing the dilutions 1 mL is transferred from the 10^0 suspension into 9 mL of dilute to give a dilution of 10^{-1}. This procedure is repeated for further dilutions using a fresh sterile pipette for each decimal dilution. The purpose of the dilutions is to ensure that the colony count per plate is in the range of 30-300 CFU for most accurate counting.*). After plating on the agar plates (Catalog number 08-757-100D, Falcon Bacteriological Petri Dishes with Lid; Corning, New York, NY, USA; Catalog number 241830, DIFCO, BD, Franklin Lakes, NJ, USA), the plates were placed overnight in an incubator at 37 °C in darkness. According to our definition in this study, each panel was labeled as 0.5 μg mL^{-1}, 1.0 μg mL^{-1} and 1.5 μg mL^{-1} of concentration, respectively. The number of surviving bacteria was determined and expressed as a percentage (%) that corresponds to the unit of CFU/mL after incubation. Data are presented as mean ± SD (n= 6).

Determination for bacteria viability rates after laser exposure

CFU counting method: MRSA or *E. coli* (OD_{600}: ~0.05) were added with materials-Ab (0.5 μg mL^{-1}) by incubating at 37 °C in darkness. After incubation, the mixture was centrifuged (approximately 1500 RCF for 20 min) and exposed to a 60-s 670-nm laser (0.07 W cm^{-2}). The pellets were with 3h of additional incubation time to process the PDT action. Before plating, dilution should be carried out in 10 folds steps using the maximum recovery dilution medium. After plating on the agar plates, the plates were placed overnight in an incubator at 37 °C in darkness. The number of surviving bacteria was determined and expressed as a percentage (%) that corresponds to the unit of CFU/mL after incubation. Data are presented as mean ± SD (n = 6).

LIVE/ DEAD kit [17]: Materials-Ab (0.5 μg mL^{-1}) was added to MRSA or *E. coli* (OD_{600}: ~0.05) with incubating at 37 °C. After incubation, the mixture was centrifuged (approximately 1500 RCF for 20 min) and exposed to a 60-s 670-nm laser (fixed output power: 0.07 W cm^{-2}). The pellets were with 3h of additional incubation time to process the PDT action. Then, the pellets were stained using a LIVE (SYTO 9, as displayed with green fluorescence) / DEAD (propidium iodide, PI, as displayed with red fluorescence) kit (Catalog number L7007, Thermo Fisher Scientific, Waltham, MA, USA) according to the instruction. The fluorescence images were obtained using the IXplore Standard microscopy coupled with the DP23 camera and analyzed with the cellSens Entry software (Olympus, Shinjuku City, Tokyo, Japan).

The viability of bacteria was quantified for antimicrobial tests, which showed nearly all material-treated bacteria to be dead after treatment. Similar viability was quantified through the CFU counting method to determine the efficient antibacterial effects of materials in PDT. Data are presented as mean ± SD (n= 6).

Uptake assay

MRSA (OD_{600}: ~0.05) were incubated with 0.5 μg mL^{-1} material-Ab. The absorbance of 0.5 μg mL^{-1} material-Ab was recorded using UV-vis spectroscopy (Abs: ~204 nm). The materials were mixed with MRSA (OD_{600}: ~0.05) at 37 °C from 1h to 10h, respectively. Then, the mixture was centrifuged (approximately 1500 RCF for 20 min) to remove excess materials, keep the supernatant, and measure the absorbance of the supernatant. The difference between the absorbance values of the collected supernatant and the original materials was estimated, thus resulting in the percentage of uptake at each time point. Data are presented as mean ± SD (n= 6).

Statistical Analysis

All analyzes were conducted in 6 repeats. Statistical analysis was performed using Graphpad Prism version 5.0a (GraphPad Software Inc. La Jolla, San Diego, CA, USA). Data were analyzed using the Student's *t*-test. Results are expressed as mean ± SD. Statistical significance was established at the level of *$p < 0.05$ (*$p < 0.05$, **$p < 0.01$, ***$p < 0.001$).

And there is no batch to batch variation for the materials in terms of photoproperties, and contrast agents.

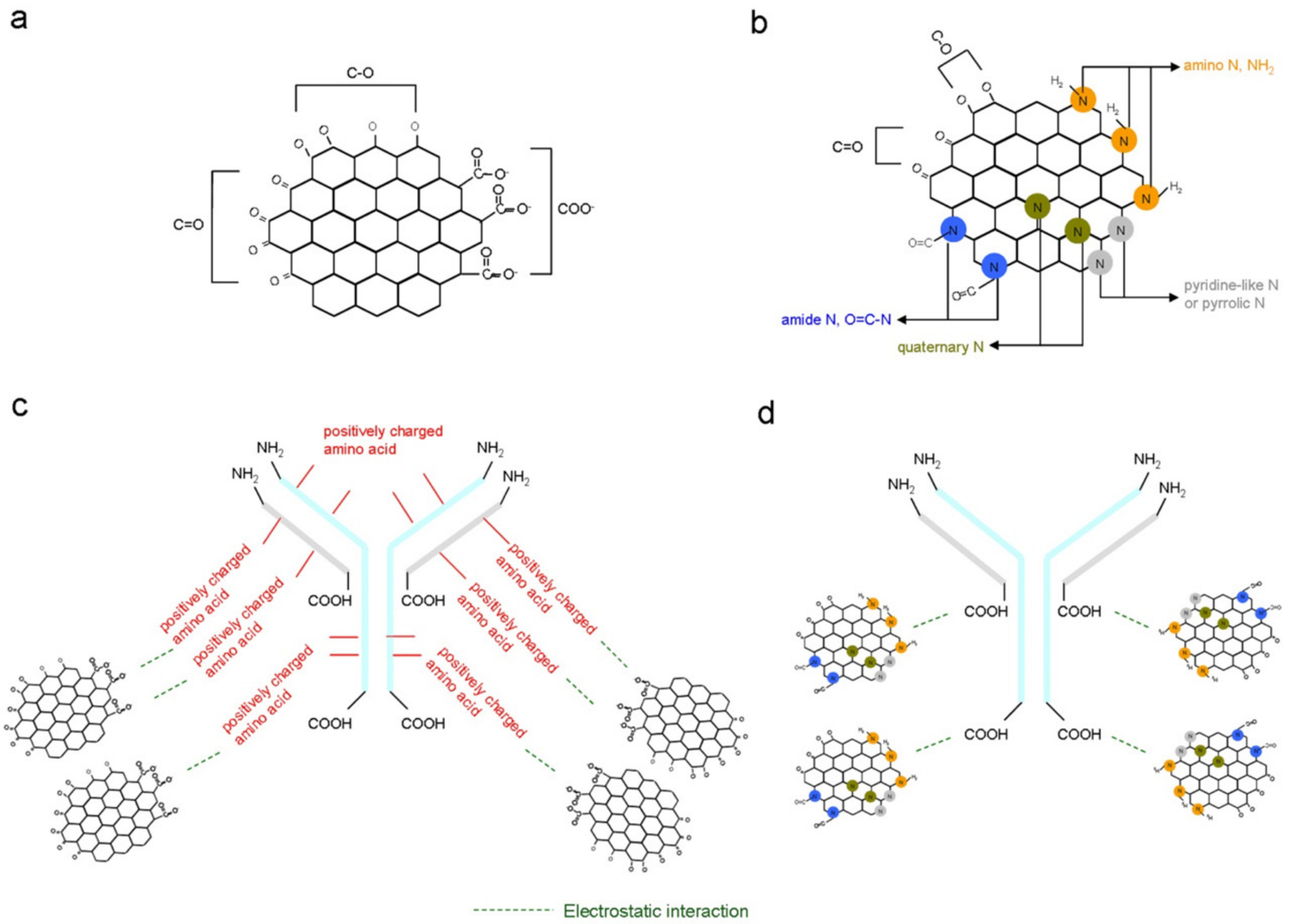

Scheme S1. The conceptual schemes of (**a**) the GQD, (**b**) the amino-N-GQD, (**c**) GQD-Ab and (**d**) amino-N-GQD-Ab.

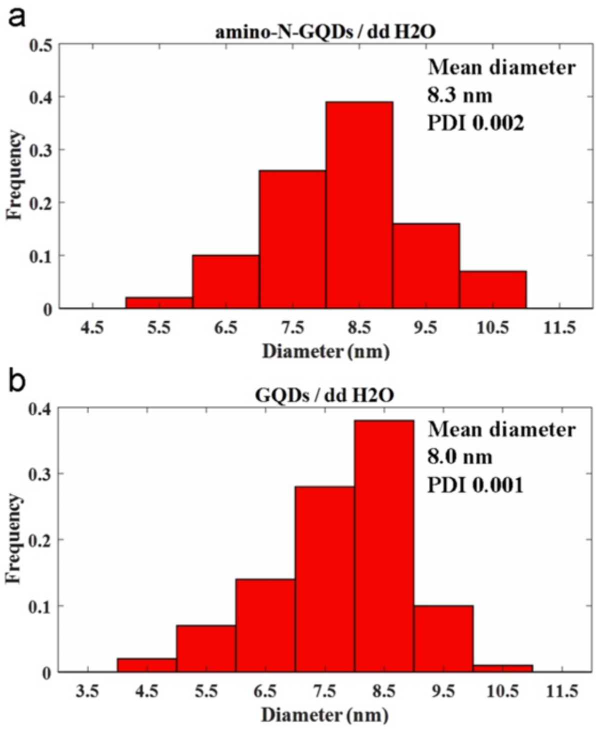

Figure S1. Quantitative size distributions of (**a**) amino-N-GQDs or (**b**) GQDs were determined by DLS. [Cell type: ZEN0112; Measurement duration: 30 number of runs, 20 run duration (sec)]. Delivered dose: 5 mg mL^{-1} material.

Raman spectroscopy was used to monitor the inelastic phonon scattering caused by the vibration of chemical bonds, which enables to calculate the size of the sp^2 domain in the amino-N-GQD and amino-N-GQD-polymers, respectively. If the mean size of the GQD-based materials > 3 nm, follow the Equation (S2) [5],

$$L_a \text{ (nm)} = (2.4 \times 10^{-10})\ \lambda_{\text{laser}}^4\ (I_D/I_G)^{-1} \tag{S2}$$

where L_a (nm) is the mean size of the sp^2 domain; λ_{Laser} is the excitation wavelength (nm); I means the intensity for the D band and G band, respectively.

However, if the mean size of the GQD-based materials < 3 nm, follow the Equation (S3) [5].

$$L_D = 0.54\ E_L^4\ (I_D/I_G) \tag{S3}$$

where is that in small-size graphene sheets with point-like defects containing sp^3-bonded carbon atoms, the mean distance between defects, L_D (nm), is generally used to represent the size of the sp^2domains $L_{D,}$ (nm); E_{Laser} is the excitation laser energy (eV) used in Raman experiments.

The Raman spectra were obtained with a 532 nm laser and were decomposed into the D band and G band by the Lorentzian function. According to the calculations based on the Equations (S2-S3), the diameter of the amino-N-GQD and GQD was 8.0 nm (compared to 8.3 ± 0.4 nm of that determined by HR-TEM) and 7.8 nm (compared to 8.0 ± 0.3 nm of that determined by HR-TEM).

Under the light illumination (not in darkness), the viabilities of materials-Ab (0.5-1.5 μg mL^{-1})-treated-MRSA or -*E. coli* according to the CFU assay (**Figure S2**). The viability was approximately 93.1% for the amino-N-GQD–Ab$_{protein\ A}$ (0.5 μg mL^{-1})-MRSA, approximately 89.3% for the amino-N-GQD–Ab$_{protein\ A}$ (1.0 μg mL^{-1})-MRSA, approximately 85.1% for the amino-N-GQD–Ab$_{protein\ A}$ (1.5 μg mL^{-1})-MRSA, approximately 94.5% for the GQD–Ab$_{protein\ A}$ (0.5 μg mL^{-1})-MRSA, approximately 87.0% for the GQD–Ab$_{protein\ A}$ (1.0 μg mL^{-1})-MRSA, and approximately 85.5% for the amino-N-GQD–Ab$_{protein\ A}$ (1.5 μg mL^{-1})-MRSA (**Figure S2a,c**). This value corresponded to an approximately 0.032, 0.019, 0.030, 0.025, 0.024, 0.033 and 0.016 log$_{10}$ reduction with size increased (**Figure S2b,d**). Indeed, the viabilities of amino-N-GQD (0.5-1.5 μg·mL^{-1})-treated MRSA decreased from approximately 7% to 15%, which was somewhat influenced by light illumination (not in the dark). A low dose (0.5 μg mL^{-1}) of amino-N-GQDs that had been incubated for 3 h with bacteria (OD$_{600}$: ~0.05) at 37 °C in the dark was employed in all subsequent experiments.

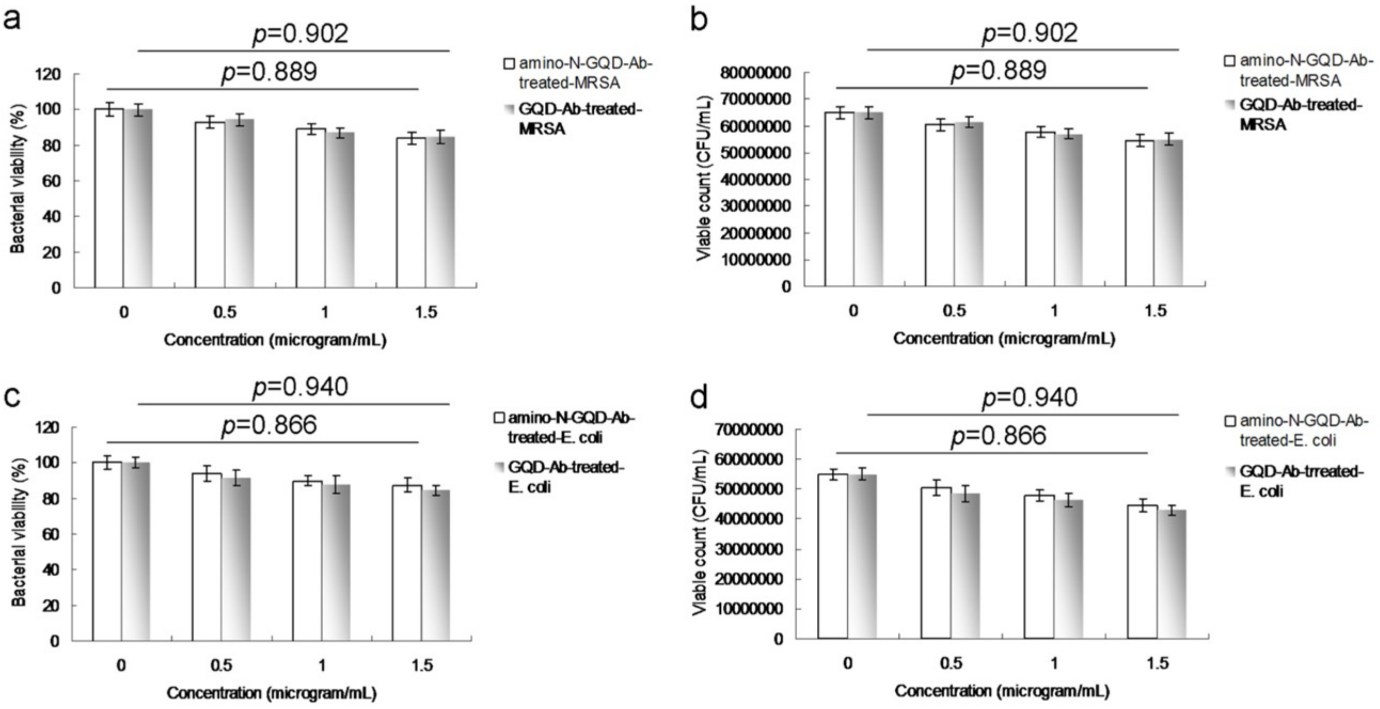

Figure S2. Numbers of surviving (**a,b**) amino-N-GQD- or GQD-Ab$_{protein\ A}$-treated-MRSA and (**c.d**) amino-N-GQD- or GQD-Ab$_{LPS}$-treated-*E. coli* were determined by CFU counting assay and are expressed as the percentage (%) for bacteria that corresponds to the unit of CFU/mL. Delivered dose: OD$_{600}$ ~ 0.05 of bacteria and 0-1.5 μg mL^{-1} material-Ab. Data are presented as mean ± SD (n = 6).For amino-N-GQD- and GQD-Ab$_{protein\ A}$-treated-MRSA with photoexcitation, (**a,b**) p= 0.899 and p= 0.902, respectively. For amino-N-GQD- and GQD-Ab$_{LPS}$-treated-*E. coli* with photoexcitation, (**c,d**) p= 0.866 and p= 0.940, respectively. *p value calculated using Student's t test (*p < 0.05, **p< 0.01, ***p< 0.001).

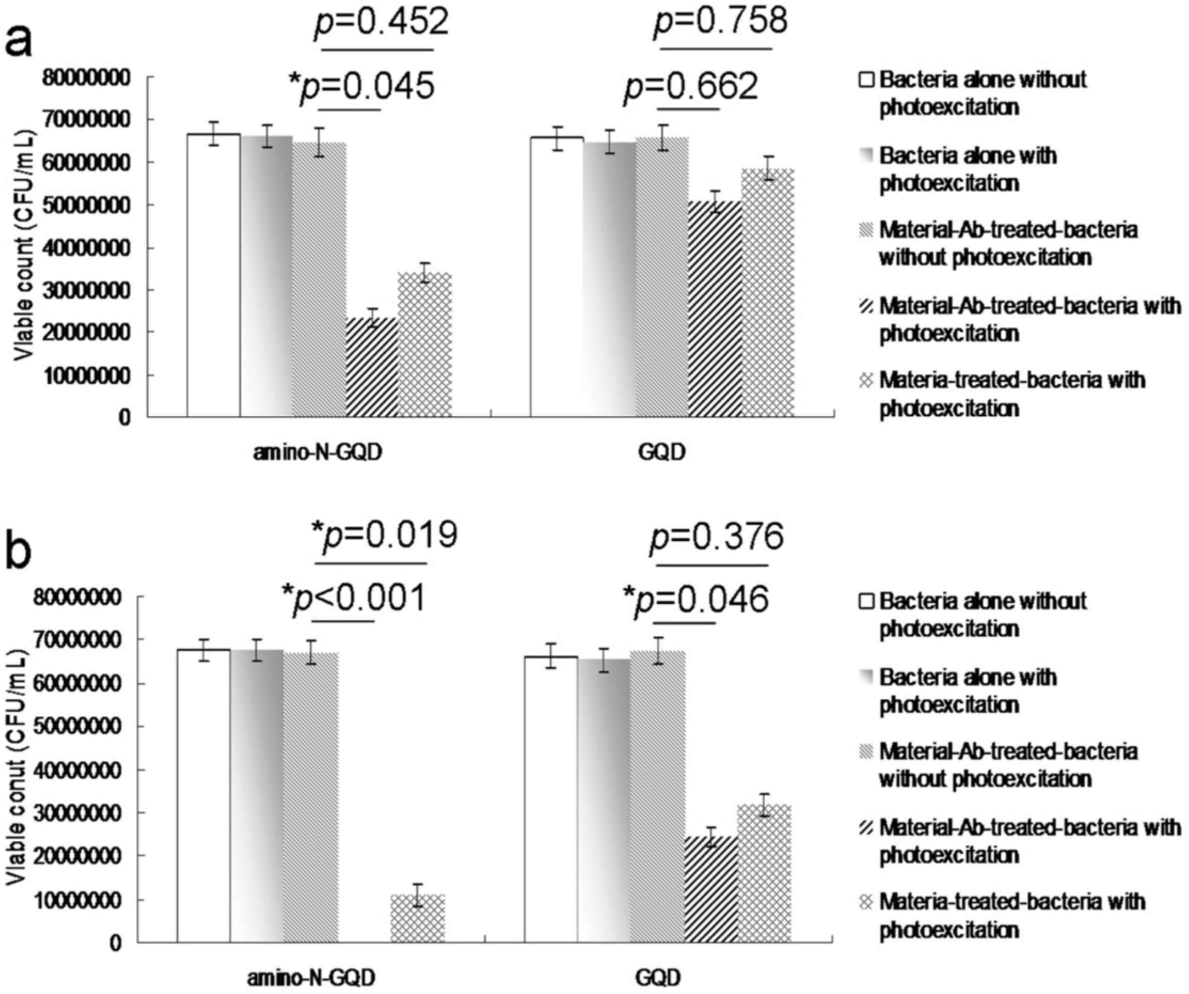

Figure S3. Number of surviving material–$Ab_{protein\ A}$-treated-MRSA via CFU assay by short excitation with a 670 nm laser (0.07 W cm^{-2}) for (**a**) 30 s and (**b**) 60 s. Delivered dose: OD_{600} ~ 0.05 of bacteria and 0.5 μg mL^{-1} material-$Ab_{protein\ A}$. Data are presented as mean ± SD (*n*= 6). For amino-N-GQD- and GQD-$Ab_{protein\ A}$-treated MRSA with photoexcitation, (**a**) *p= 0.045 and p= 0.662, (**b**) ***p< 0.001 and *p= 0.046. For amino-N-GQD- and GQD-treated MRSA with photoexcitation, (**a**) p= 0.425 and p= 0.758 and (**b**) *p= 0.019 and p= 0.376, respectively. *p value calculated using Student's *t* test (*p < 0.05, **p< 0.01, ***p< 0.001).

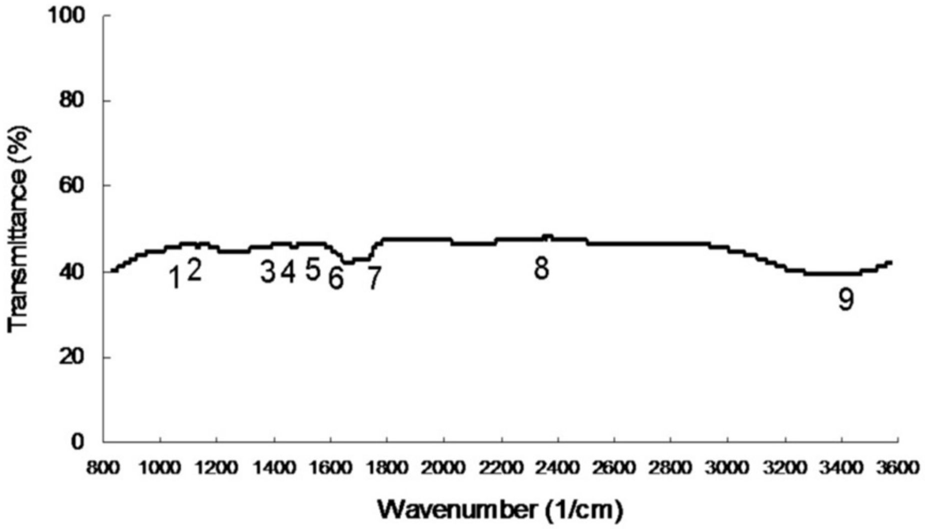

Figure S4. FTIR spectrum of GQD. The characteristic bands of GQD for C–O stretching at approximately 1018 cm^{-1} (band 1), phenolic C-OH stretching at approximately 1179 cm^{-1} (band 2), symmetric carboxylate $C(=O)_2^-$ stretching at approximately 1385 cm^{-1} (band 3), tertiary alcoholic C–OH bending at approximately 1437 cm^{-1} (band 4), asymmetric carboxylate $C(=O)_2^-$ stretching at approximately 1588 cm^{-1} (band 5), C=C ring stretching at approximately 1615 cm^{-1} (band 6), C=O stretching at approximately 1792 cm^{-1} (band 7), broad O–H stretching at approximately 2386 cm^{-1} (band 8), and cyclic alkanes, C–H stretching, intermolecular hydrogen bonded and O–H stretching at approximately 3410 cm^{-1} (band 9). Delivered dose: 0.5 μg mL^{-1} for material.

The GQD exhibited favorable biocompatibility according to the CFU assay (**Figure S5**).

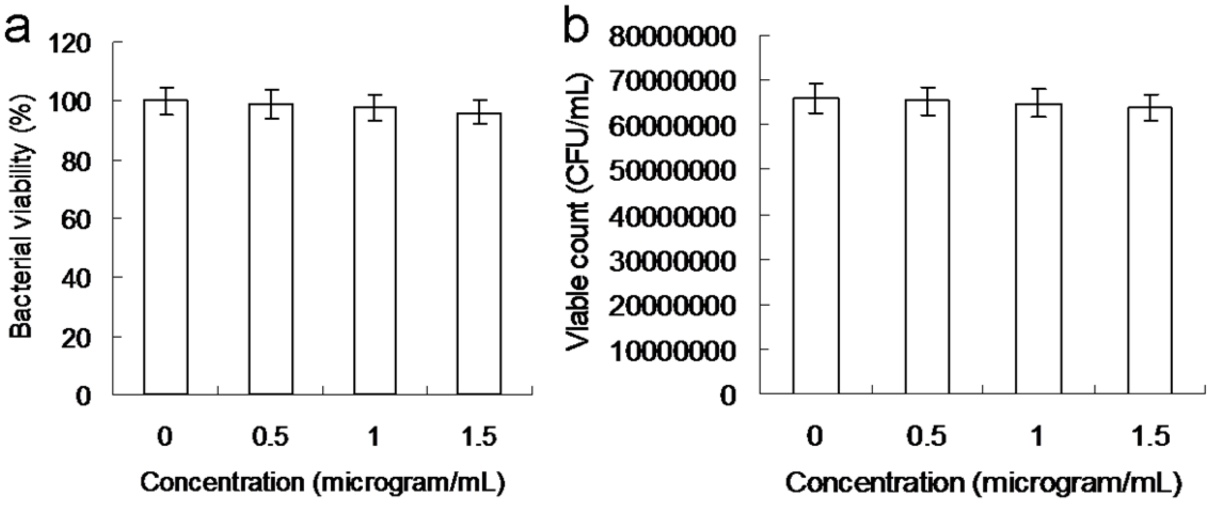

Figure S5. Number of surviving (**a**) GQD-$Ab_{protein\ A}$-treated-MRSA was determined by CFU counting assay and are expressed as the percentage (%) for (**b**) MRSA that corresponds to the unit of CFU/mL. Delivered dose: OD_{600} ~ 0.05 of bacteria and 0-1.5 µg mL^{-1} material-$Ab_{protein\ A}$. Data are presented as mean ± SD (n = 6).

Table S1. Comparison of characteristics between GQD and amino-N-GQD.

Instrument	GQD	amino-N-GQD
TEM	mean lateral size~8.0 ± 0.3 nm	mean lateral size~8.3 ± 0.4 nm
XRD	$2\theta = 24.3^{\circ}$	$2\theta = 24.3^{\circ}$
Raman	D band:1383 cm^{-1}, G band: 1607 cm^{-1}, I_D/I_G: 0.89	D band:1384 cm^{-1}, G band: 1606 cm^{-1}, I_D/I_G: 0.90
UV-vis	211 nm ($\pi \rightarrow \pi^*$ transition of aromatic C=C bonds) and 312 nm ($n \rightarrow \pi^*$ transition of C=O shoulder)	212 nm ($\pi \rightarrow \pi^*$ transition of aromatic C=C bonds) and 315 nm ($n \rightarrow \pi^*$ transition of C=O shoulder and C–N)
FTIR	C–O stretching~1018 cm^{-1}, phenolic C–OH stretching~1179 cm^{-1}, symmetric carboxylate $C(=O)_2^-$ stretching~1385 cm^{-1}, tertiary alcohol C–OH bending~1437 cm^{-1}, asymmetric carboxylate $C(=O)_2^-$ stretching~1588 cm^{-1}, C=C ring stretching~1615 cm^{-1}, C=O stretching~1792 cm^{-1}, broad O–H stretching~2386 cm^{-1}, and cyclic alkanes, C–H stretching, intermolecular hydrogen bonded and O–H stretching~3410 cm^{-1}	C–O stretching~1023 cm^{-1}, C–N stretching~1150 cm^{-1}, N–C=O stretching~1212 cm^{-1}, tertiary alcohol C–OH stretching~1448 cm^{-1}, C=C ring stretching~1611 cm^{-1}, N–H bonding and amide stretching~1786 cm^{-1}, C=O stretching~1833 cm^{-1}, N–H stretching~2971 cm^{-1}, and N–H vibration~ 3455 cm^{-1}
XPS	deconvoluted C(1s) spectrum: C–C/C=C (285.8 eV, C–O (286.9 eV), C=O (288.1 eV), and O=C–O (289.0 eV) bonds	deconvoluted C(1s) spectrum: C–C/C=C (285.0 eV, C–N (286.6 eV), C–O (287.1 eV), and carbonyl (C=O, 288.0 eV) bonds; deconvoluted N(1s) spectrum: pyridinic N (398.3 eV), amino N (NH2, 398.9 eV), pyrrolic N (399.6 eV), quaternary N (400.4 eV), and amide N (O=C–N, 401.2 eV

The materials exhibited favorable biocompatibility according to the CFU assay (**Figure S6)**

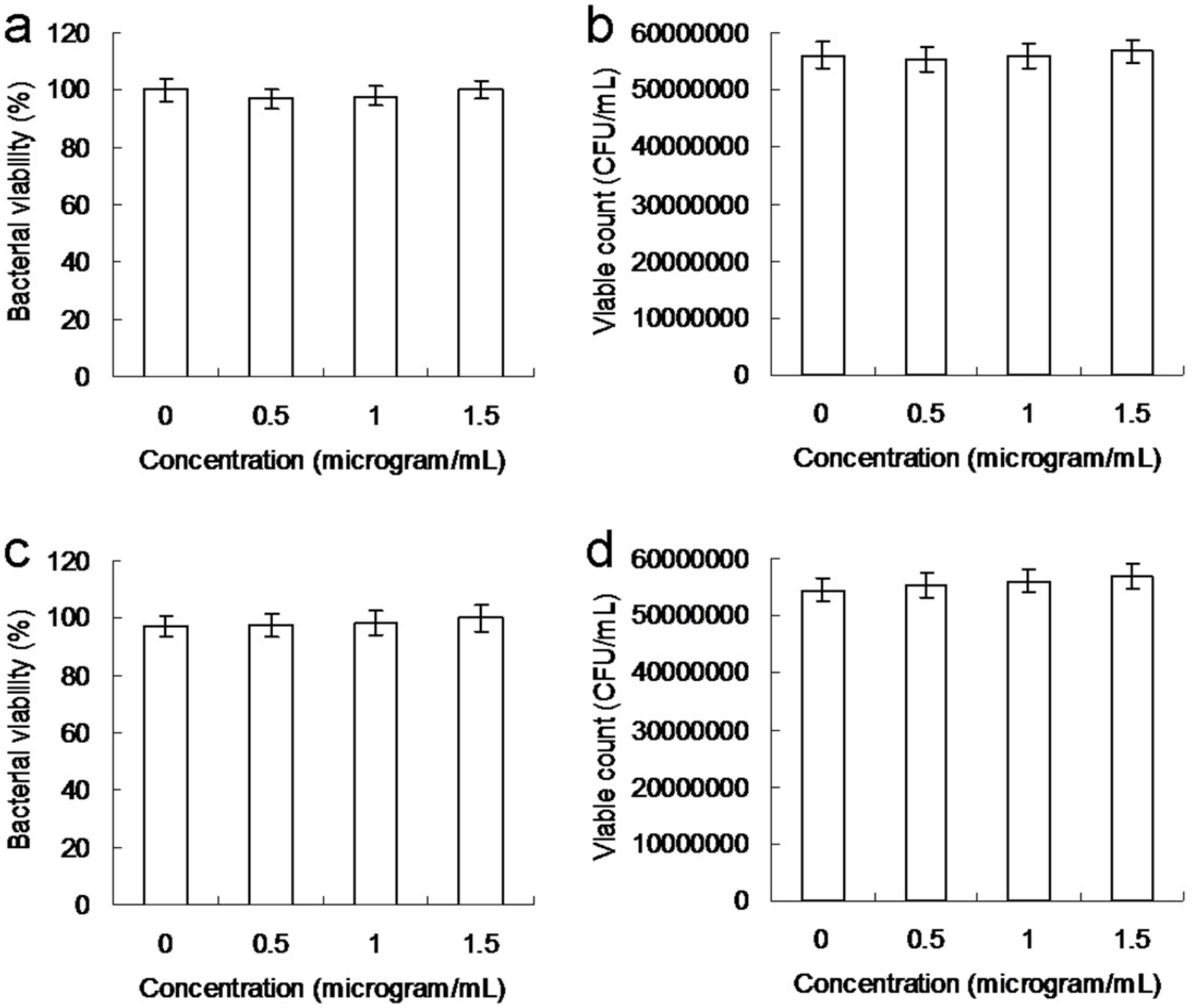

Figure S6. Number of surviving (**a**) GQD- and (**c**) amino-N-GQD-Ab_{LPS}-treated-*E. coli* was determined by CFU counting assay and are expressed as the percentage (%) for (**b**) and (**d**) *E. coli* that corresponds to the unit of CFU/mL. Delivered dose: OD_{600} ~ 0.05 of bacteria and 0-1.5 μg mL^{-1} material-Ab_{LPS}. Data are presented as mean ± SD (n = 6).

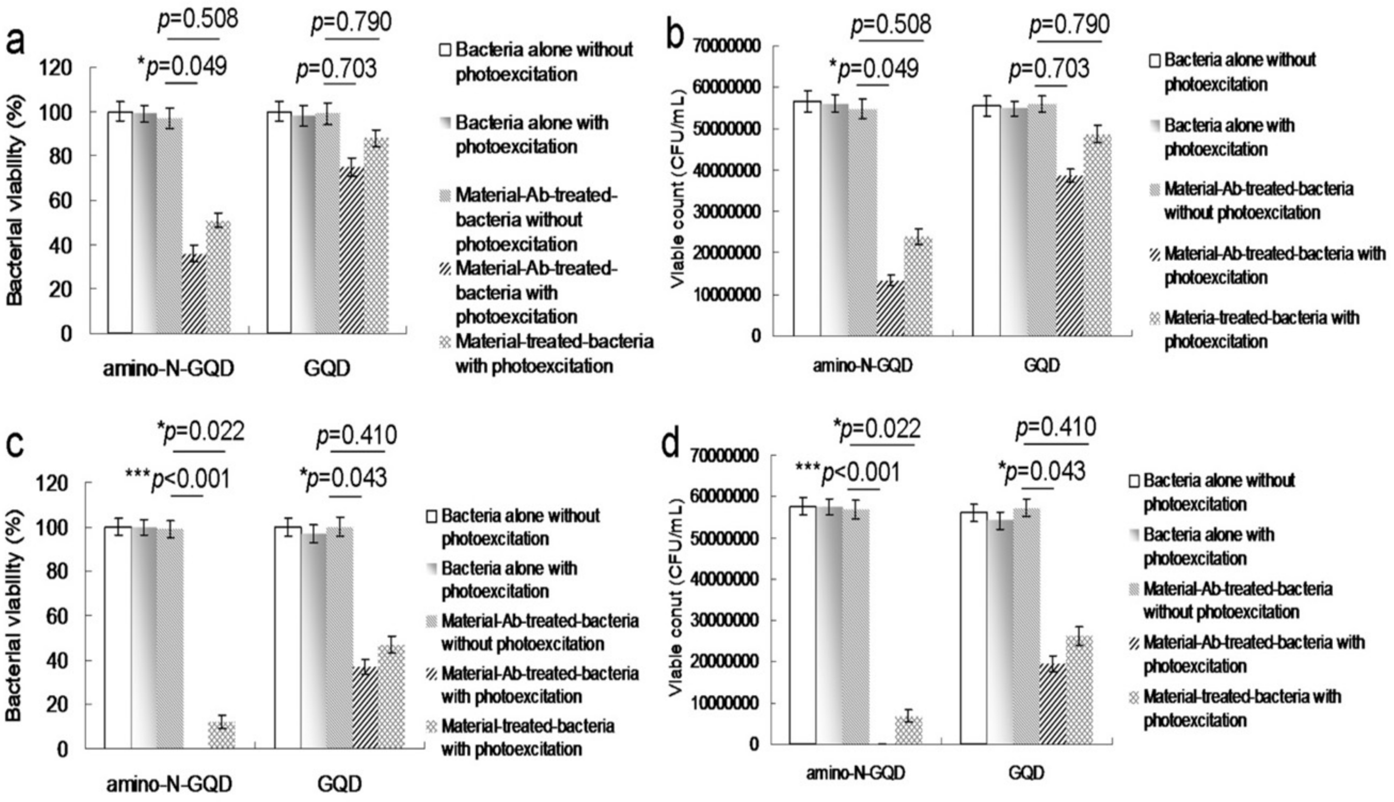

Figure S7. Number of surviving material-Ab_{LPS}-treated-*E. coli* via CFU assay by short excitation with a 670 nm laser (0.07 W cm^{-2}) for (**a**) 30 s and (**b**) 60 s. Delivered dose: OD_{600} ~ 0.05 of bacteria and 0.5 μg mL^{-1} material-$Ab_{protein\ A}$. Data are presented as mean ± SD (n= 6). For amino-N-GQD- and GQD- Ab_{LPS}-treated-*E. coli* with photoexcitation, (**a,b**) *p= 0.049 and p= 0.703, (**c,d**) ***p< 0.001 and *p= 0.043. For amino-N-GQD- and GQD-treated-*E. coli* with photoexcitation, (**a,b**) p= 0.508 and p= 0.790 ,and (**c,d**) *p= 0.022 and p= 0.410, respectively. *p value calculated using Student's t test (*p < 0.05, **p< 0.01, ***p< 0.001).

Table S2. Amount of ROS generated by conducting photoexcitation (0.07 W cm^{-2}; Ex: 670 nm) and by using materials was maintained in the dark and monitored. Delivered dose: 0.5 µg mL^{-1} for material. Data are presented as mean ± SD (n = 6) [5,15,17,20].

			$^1O_2$ (by SOSG)[c]				
	Negative control[ac]	ROS neutralization[abc]	Positive control[cd]	amino-N-GQD	ROS neutralization[bc]	GQD	ROS neutralization[bc]
30-s photoexcitation	238±9	240±12	1997±88	1406±45	238±10	847±22	240±10
60-s photoexcitation	240±11	241±12	2735±146	2159±99	241±10	1203±37	241±11

			$^1O_2$ (by *t*-MVP)[e]				
	Negative control[ae]	ROS neutralization[abe]	Positive control[de]	amino-N-GQD	ROS neutralization[be]	GQD	ROS neutralization[be]
30-s photoexcitation	325±20	324±18	6073±149	4868±112	324±20	2766±74	325±19
60-s photoexcitation	323±21	324±20	8605±180	5990±145	325±21	3801±98	325±20-

			$O_2\bullet^-$ (by XTT)[f]				
	Negative control[af]	ROS neutralization[abf]	Positive Control[df]	amino-N-GQD	ROS neutralization[bf]	GQD	ROS neutralization[bf]
30-s photoexcitation	0	0	1.17±0.09	0.99±0.08	0.02±0.01	0.68±0.07	0.02±0.01
60-s photoexcitation	0	0	2.05±0.16	1.76±0.14	0.03±0.01	1.05±0.08	0.03±0.02

			$O_2\bullet^-$ (by GSH)[g]				
	Negative control[ag]	ROS neutralization[abg]	Positive Control[dg]	amino-N-GQD	ROS neutralization[bg]	GQD	ROS neutralization[bg]
30-s photoexcitation	0	0	79.5±3.2%	68.9±2.9%	0.3±0.1%	45.2±1.7%	0.2±0.1%
60-s photoexcitation	0	0	99.8±4.7%	90.3±4.0%	0.3±0.2%	59.2±2.0%	0.3±0.1%

[a]Negative control: only treat using reagent and laser radiation without using any material (0 µg mL^{-1}).

[b]ROS neutralization: includes nanomaterial treatment, laser irradiation and 30 ppm of antioxidant α-Tocopherol / methyl linoleate.

[c]The SOSG reagent (Ex/Em: 488/525 nm) has a specific reactivity to generate fluorescence that is recorded using a PL spectrometer.

[d]Positive control: treatment of 50 µM *tert*-butyl hydroperoxide (TBHP, CAS number 75-91-2, Merck & Co., Kenilworth, NJ, USA) and laser irradiation.

[e]*t*-MVP (Ex/Em: 352/465 nm) can react with $^1O_2$, and form a dioxetane intermediate that generates fluorescence upon decomposition to 1-pyrenecarboxaldehyde. The process is monitored using a PL spectrometer.

[f]XTT can interact with $O_2^{\cdot-}$ and produce XTT-formazan that generates strong absorption (wavelength: 470 nm).

[g]GSH containing a thiol-tripeptide can prevent damages to cellular or bacterial components caused by stress of oxidation. The thiol group from GSH can be oxidized to the disulfide bond, thus converting GSH to glutathione disulfide. GSH oxidation was used to determine the generated $O_2^{\cdot-}$. Loss of GSH (%) = (difference between of the absorbance of the sample and negative control / absorbance of negative control) × 100 %.

Table S3. Amount of ROS generated by conducting photoexcitation (0.07 W cm^{-2}; Ex: 670 nm) and by using materials-Ab-treated-bacteria was maintained in the dark and monitored. Delivered dose: OD_{600} = ~0.05 for *E. coli* and 0.5 μg mL^{-1} for material. Data are presented as mean ± SD (*n*= 6) [5,15,17,20].

			$^1O_2$ (by SOSG)[c]				
	Negative control[ac]	ROS neutralization[abc]	Positive control[cd]	amino-N-GQD	ROS neutralization[bc]	GQD	ROS neutralization[bc]
30-s photoexcitation	243±9	240±11	2007±85	1031±40	241±13	509±21	242±10
60-s photoexcitation	244±12	242±12	2704±139	1722±51	241±14	882±33	244±9
			$^1O_2$ (by *t*-MVP)[e]				
	Negative control[ae]	ROS neutralization[abe]	Positive control[de]	amino-N-GQD	ROS neutralization[be]	GQD	ROS neutralization[be]
30-s photoexcitation	320±18	320±16	6024±138	4116±104	321±20	2160±57	322±19
60-s photoexcitation	324±20	323±20	8566±171	5350±130	320±22	3014±81	325±222
			$O_2\bullet^-$ (by XTT)[f]				
	Negative control[af]	ROS neutralization[abf]	Positive Control[df]	amino-N-GQD	ROS neutralization[bf]	GQD	ROS neutralization[bf]
30-s photoexcitation	0	0	1.12±0.07	0.87±0.08	0.01±0.01	0.58±0.05	0.02±0.01
60-s photoexcitation	0	0	1.96±0.13	1.42±0.12	0.02±0.01	0.86±0.08	0.02±0.01
			$O_2\bullet^-$ (by GSH)[g]				
	Negative control[ag]	ROS neutralization[abg]	Positive Control[dg]	amino-N-GQD	ROS neutralization[bg]	GQD	ROS neutralization[bg]
30-s photoexcitation	0	0	79.6±3.0%	60.5±2.0%	0.2±0.1%	36.6±1.3%	0.3±0.1%
60-s photoexcitation	0	0	99.3±4.1%	84.8±2.9%	0.1±0.1%	52.5±2.0%	0.2±0.1%

[a]Negative control: only treat using reagent and laser radiation without using any material (0 μg mL^{-1}).

[b]ROS neutralization: includes nanomaterial treatment, laser irradiation and 30 ppm of antioxidant *α*-Tocopherol/methyl linoleate.

[c]The SOSG reagent (Ex/Em: 488/525 nm) has a specific reactivity to generate fluorescence that is recorded using a PL spectrometer.

[d]Positive control: treatment of 50 μM *tert*-butyl hydroperoxide and laser irradiation.

[e]*t*-MVP (Ex/Em: 352/465 nm) can react with $^1O_2$, and form a dioxetane intermediate that generates fluorescence upon decomposition to 1-pyrenecarboxaldehyde. The process is monitored using a PL spectrometer.

[f]XTT can interact with $O_2^{\cdot-}$ and produce XTT-formazan that generates strong absorption (wavelength: 470 nm).

[g]GSH containing a thiol-tripeptide can prevent damages to cellular or bacterial components caused by stress of oxidation. The thiol group from GSH can be oxidized to the disulfide bond, thus converting GSH to glutathione disulfide. GSH oxidation was used to determine the generated $O_2^{\cdot-}$. Loss of GSH (%) = (difference

between of the absorbance of the sample and negative control / absorbance of negative control) × 100 %

Table S4. Amount of ROS generated by conducting photoexcitation (0.07 W cm^{-2}; Ex: 670 nm) and by using materials-treated-bacteria was maintained in the dark and monitored. Delivered dose: OD_{600} = ~0.05 for MRSA and 0.5 μg mL^{-1} for material. Data are presented as mean ± SD (*n* = 6) [5,15,17,20].

			$^1O_2$ (by SOSG)[c]				
	Negative control[ac]	ROS neutralization[abc]	Positive control[cd]	amino-N-GQD	ROS neutralization[bc]	GQD	ROS neutralization[bc]
30-s photoexcitation	235±9	236±12	1943±77	852±30	235±10	413±18	235±11
60-s photoexcitation	236±10	235±12	2600±134	1455±47	236±10	996±35	236±12

			$^1O_2$ (by *t*-MVP)[e]				
	Negative control[ae]	ROS neutralization[abe]	Positive control[de]	amino-N-GQD	ROS neutralization[be]	GQD	ROS neutralization[be]
30-s photoexcitation	322±18	323±18	6004±136	3790±98	323±19	1805±48	324±20
60-s photoexcitation	321±20	322±10	8502±170	4980±114	323±21	2812±70	323±20-

			$O_2 \bullet^-$ (by XTT)[f]				
	Negative control[af]	ROS neutralization[abf]	Positive Control[df]	amino-N-GQD	ROS neutralization[bf]	GQD	ROS neutralization[bf]
30-s photoexcitation	0	0	1.10±0.06	0.72±0.05	0.02±0.01	0.43±0.05	0.02±0.01
60-s photoexcitation	0	0	1.93±0.12	1.19±0.07	0.03±0.02	0.72±0.06	0.03±0.01

			$O_2 \bullet^-$ (by GSH)[g]				
	Negative control[ag]	ROS neutralization[abg]	Positive Control[dg]	amino-N-GQD	ROS neutralization[bg]	GQD	ROS neutralization[bg]
30-s photoexcitation	0	0	77.9±3.0%	54.2±2.5%	0.3±0.1%	29.6±1.2%	0.2±0.1%
60-s photoexcitation	0	0	98.5±4.2%	75.3±2.7%	0.3±0.1%	45.1±1.6%	0.3±0.1%

[a]Negative control: only treat using reagent and laser radiation without using any material (0 μg mL^{-1}).

[b]ROS neutralization: includes nanomaterial treatment, laser irradiation and 30 ppm of antioxidant α-Tocopherol/methyl linoleate.

[c]The SOSG reagent (Ex/Em: 488/525 nm) has a specific reactivity to generate fluorescence that is recorded using a PL spectrometer.

[d]Positive control: treatment of 50 μM TBHP and laser irradiation.

[e]*t*-MVP (Ex/Em: 352/465 nm) can react with $^1O_2$, and form a dioxetane intermediate that generates fluorescence upon decomposition to 1-pyrenecarboxaldehyde. The process is monitored using a PL spectrometer.

[f]XTT can interact with $O_2^{\cdot-}$ and produce XTT-formazan that generates strong absorption (wavelength: 470 nm).

[g]GSH containing a thiol-tripeptide can prevent damages to cellular or bacterial components caused by stress of oxidation. The thiol group from GSH can be oxidized to the disulfide bond, thus converting GSH to glutathione disulfide. GSH oxidation was used to determine the generated $O_2^{\cdot-}$. Loss of GSH (%) = (difference between of the absorbance of the sample and negative control / absorbance of negative control) × 100 %.

Table S5. Amount of ROS generated by conducting photoexcitation (0.07 W cm^{-2}; Ex: 670 nm) and by using materials-treated-bacteria was maintained in the dark and monitored. Delivered dose: OD_{600} = ~0.05 for *E. coli* and 0.5 μg mL^{-1} for material. Data are presented as mean ± SD (*n* = 6) [5,15,17,20].

			$^1O_2$ (by SOSG)[c]				
	Negative control[ac]	ROS neutralization[abc]	Positive control[cd]	amino-N-GQD	ROS neutralization[bc]	GQD	ROS neutralization[bc]
30-s photoexcitation	238±10	235±11	1989±82	894±34	235±9	422±17	233±10
60-s photoexcitation	237±13	235±12	2643±137	1482±50	237±11	1013±36	234±11
			$^1O_2$ (by *t*-MVP)[e]				
	Negative control[ae]	ROS neutralization[abe]	Positive control[de]	amino-N-GQD	ROS neutralization[be]	GQD	ROS neutralization[be]
30-s photoexcitation	320±16	320±15	6018±140	3803±100	320±12	1814±50	320±14
60-s photoexcitation	319±19	320±12	8515±163	5005±115	319±19	2802±67	318±14-
			O_2 • $^-$ (by XTT)[f]				
	Negative control[af]	ROS neutralization[abf]	Positive Control[df]	amino-N-GQD	ROS neutralization[bf]	GQD	ROS neutralization[bf]
30-s photoexcitation	0	0	1.05±0.04	0.70±0.06	0.02±0.01	0.40±0.05	0.01±0.01
60-s photoexcitation	0	0	1.90±0.11	1.15±0.05	0.02±0.02	0.67±0.04	0.01±0.01
			O_2 • $^-$ (by GSH)[g]				
	Negative control[ag]	ROS neutralization[abg]	Positive Control[dg]	amino-N-GQD	ROS neutralization[bg]	GQD	ROS neutralization[bg]
30-s photoexcitation	0	0	76.4±2.6%	52.9±2.2%	0.1±0.1%	28.1±0.9%	0.2±0.1%
60-s photoexcitation	0	0	98.0±4.1%	74.5±2.3%	0.2±0.1%	44.2±1.5%	0.2±0.1%

[a]Negative control: only treat using reagent and laser radiation without using any material (0 μg mL^{-1}).

[b]ROS neutralization: includes nanomaterial treatment, laser irradiation and 30 ppm of antioxidant α-Tocopherol/methyl linoleate.

[c]The SOSG reagent (Ex/Em: 488/525 nm) has a specific reactivity to generate fluorescence that is recorded using a PL spectrometer.

[d]Positive control: treatment of 50 μM TBHP and laser irradiation.

[e]*t*-MVP (Ex/Em: 352/465 nm) can react with $^1O_2$, and form a dioxetane intermediate that generates fluorescence upon decomposition to 1-pyrenecarboxaldehyde. The process is monitored using a PL spectrometer.

[f]XTT can interact with $O_2^{\cdot-}$ and produce XTT-formazan that generates strong absorption (wavelength: 470 nm).

[g]GSH containing a thiol-tripeptide can prevent damages to cellular or bacterial components caused by stress of oxidation. The thiol group from GSH can be oxidized to the disulfide bond, thus converting GSH to glutathione disulfide. GSH oxidation was used to determine the generated $O_2^{\cdot-}$. Loss of GSH (%) = (difference between of the absorbance of the sample and negative control / absorbance of negative control) × 100 %.

Table S6. Stability of materials in the physiological environments was determined by DLS. [Cell type: ZEN0112; Measurement duration: 30 number of runs, 20 run duration (sec)]. Delivered dose: 5 mg mL^{-1} material.

	ddH_2O	1X PBS	MRSA BHI broth
newly prepared materials			
amino-N-GQDs	8.4 nm	8.5 nm	8.5 nm
GQDs	8.1 nm	8.2 nm	8.2 nm
As-prepared materials stayed in different physiological environments for 8 weeks			
amino-N-GQDs	8.5 nm	8.5 nm	8.5 nm
GQDs	8.2 nm	8 nm	8.2 nm

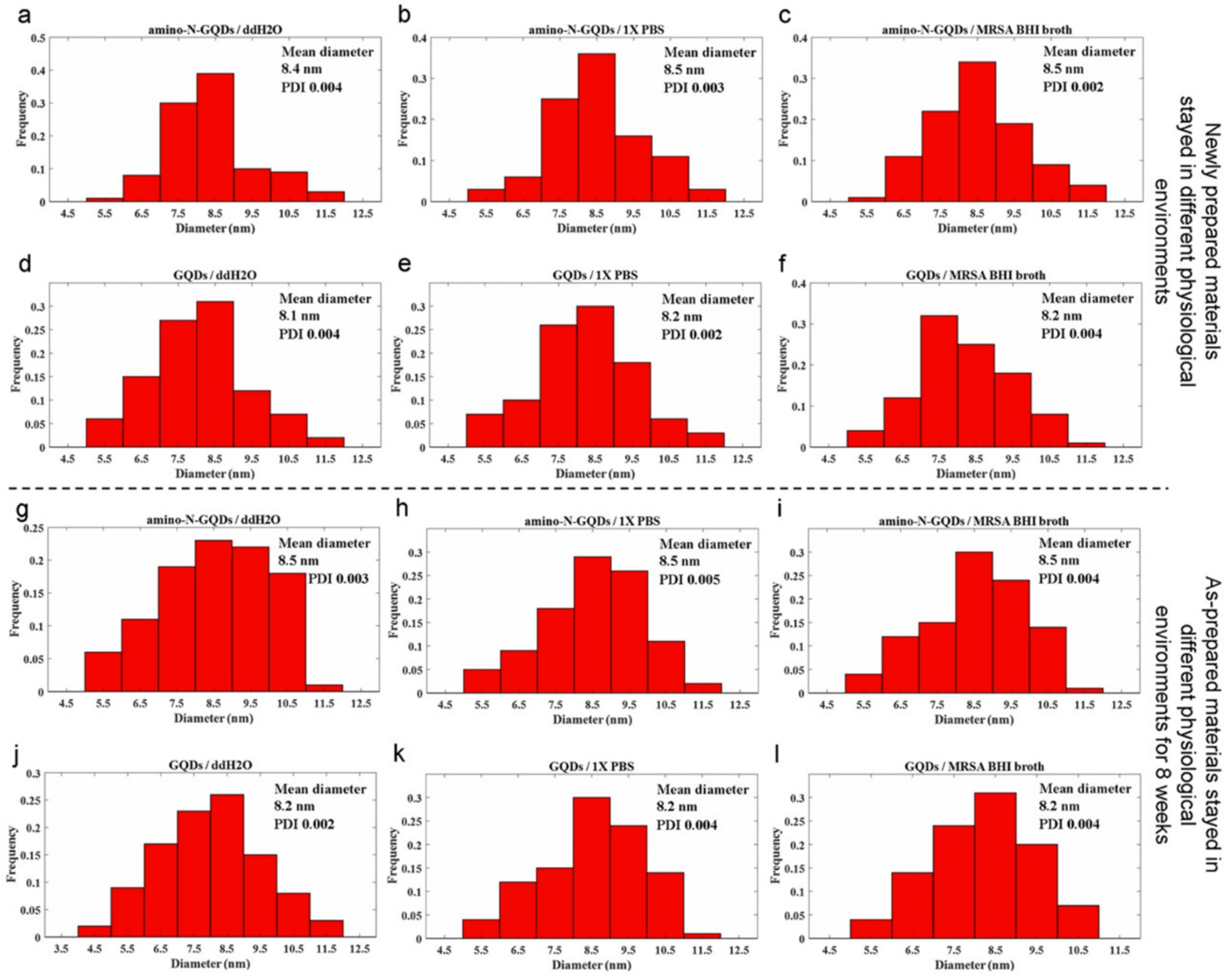

Figure S8. Stability of materials in the physiological environments was determined by DLS: newly prepare (**a-c**) amino-N-GQDs and (**d-f**) GQDs in ddH$_2$O, 1X PBS, MRSA BHI broth, respectively; as-prepared (**g-i**) amino-N-GQDs and (**j-l**) GQDs in ddH$_2$O, 1X PBS, MRSA BHI broth for 8 weeks. [Cell type: ZEN0112; Measurement duration: 30 number of runs, 20 run duration (sec)]. Delivered dose: 5 mg mL^{-1}.

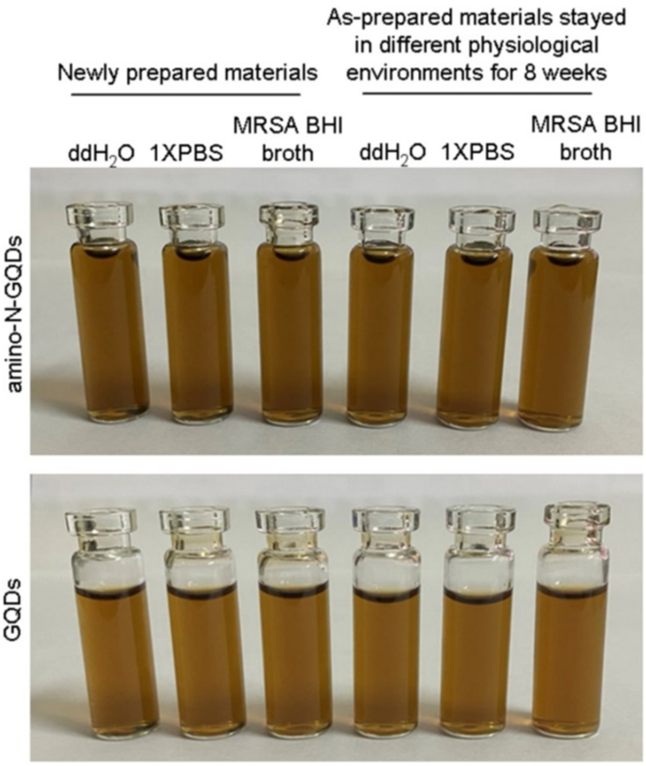

Figure S9. Stability of materials in the physiological environments was determined by DLS. Delivered dose: 5 mg mL^{-1}.

Table S7. Antibody coating efficiency and zeta potential of materials [Cell type: DTS1060C; Measurement duration: 30 number of runs, 20 run duration (sec)]. Delivered dose: 5 mg mL^{-1} material.

	1XPBS	MRSA BHI broth	*E. coli* LB broth
Amino-N-GQD-$Ab_{protein\ A}$	12.0% / 13.7 mV	10.7% / 12.5 mV	
GQD-$Ab_{proteinA}$	10.9% / 13.2 mV	10.1% / 11.4 mV	
Amino-N-GQD-Ab_{LPS}	13.1% / 14.5 mV		12.2% / 13.9 mV
GQD-Ab_{LPS}	12.5% / 13.0 mV		11.0% / 12.6 mV
